# Iron-Reversible Bactericidal Activity of Marine-Derived *Aspergillus ostianus* Hydroxamate Pyrazinones Against Replicating and Hypoxia-Induced Non-Replicating *Mycobacterium smegmatis*

**DOI:** 10.3390/md24070236

**Published:** 2026-07-03

**Authors:** Muhammad Azhari, Shinnosuke Isshiki, Riku Horinouchi, Marlia Singgih, Masayoshi Arai, Afrillia Nuryanti Garmana, Rika Hartati, Yuni Elsa Hadisaputri, Nunung Yuniarti, Elin Julianti

**Affiliations:** 1 Department of Pharmacochemistry, School of Pharmacy, Institut Teknologi Bandung, Bandung 40132, Indonesia; mazhari@itb.ac.id (M.A.); marlia@itb.ac.id (M.S.); 2 Laboratory of Natural Products for Drug Discovery, Graduate School of Pharmaceutical Sciences, The University of Osaka, Suita 565-0871, Japanaraim@phs.osaka-u.ac.jp (M.A.); 3 Department of Pharmacology and Clinical Pharmacy, School of Pharmacy, Institut Teknologi Bandung, Bandung 40132, Indonesia; afrillia@itb.ac.id; 4 Department of Pharmaceutical Biology, School of Pharmacy, Institut Teknologi Bandung, Bandung 40132, Indonesia; rikahar@itb.ac.id; 5 Department of Biological Pharmacy, Faculty of Pharmacy, Universitas Padjadjaran, Sumedang 45363, Indonesia; yuni.elsa@unpad.ac.id; 6 Department of Pharmacology and Clinical Pharmacy, Faculty of Pharmacy, Universitas Gadjah Mada, Yogyakarta 55281, Indonesia; nunung@mail.ugm.ac.id

**Keywords:** antimycobacterial, *Mycobacterium smegmatis*, non-replicating persistence, hypoxia, hydroxamate pyrazinones, iron sequestration, marine-derived *Aspergillus*

## Abstract

Tuberculosis therapy is prolonged partly because dormant subpopulations of *Mycobacterium tuberculosis* show reduced susceptibility to first-line drugs. Therefore, agents active against both replicating and non-replicating mycobacteria remain important to explore. Here, we investigated secondary metabolites from the Indonesian marine-derived fungus *Aspergillus ostianus* for activity against *Mycobacterium smegmatis*, a BSL-1 mycobacterial model, under aerobic and hypoxia-induced non-replicating conditions, and examined the underlying mechanism. Bioassay-guided fractionation and spectroscopic analysis identified three hydroxamate pyrazinones: neohydroxyaspergillic acid (NHAA), hydroxyaspergillic acid (HAA), and neoaspergillic acid (NAA). The MIC values were 1.56 µg/mL for NHAA and 3.13 µg/mL for HAA and NAA under both aerobic and hypoxic atmospheres. Time-kill kinetics showed ≥3-log10 CFU reductions within 24–72 h at 4–8× MIC under aerobic conditions and within 48–96 h at 4–8× MIC under hypoxia, with no regrowth at the final sampling point. Scanning electron microscopy and release of UV-absorbing intracellular material at OD_260_/OD_280_ were consistent with envelope disruption in both atmospheres. Antimycobacterial activity was attenuated in a concentration-dependent manner by exogenous Fe^3+^ and was reversed at 100 µM FeCl_3_, whereas isoniazid activity was unaffected, supporting iron-reversible and pyrazinone-specific killing. Together with the established Fe^3+^-binding hydroxamate pharmacophore shared by this compound class, these findings support iron sequestration as a plausible mechanism and identify fungal hydroxamate pyrazinones as scaffolds that retain bactericidal activity against hypoxia-adapted non-replicating mycobacteria, warranting further evaluation in *M. tuberculosis* models.

## 1. Introduction

Tuberculosis remains difficult to control due to the ability of *Mycobacterium tuberculosis* to enter a dormant, non-replicating state under unfavorable conditions such as hypoxia and nutrient deprivation [1]. This phenotype is tolerant to many first-line antibiotics that predominantly target actively replicating bacilli [2], thereby necessitating prolonged multidrug regimens to eliminate both active and dormant populations [3]. However, the complexity and duration of treatment often result in poor adherence, relapse, and drug resistance [4,5]. Isoniazid and ethambutol are active mainly against replicating bacilli (active state), while rifampicin and pyrazinamide show some efficacy against non-replicating forms (dormant state). Although pyrazinamide contributes sterilizing activity during the intensive phase, it is generally not included in the continuation phase of standard drug-susceptible tuberculosis regimens, partly because toxicity limits prolonged use [6,7]. Thus, standard regimens require a prolonged continuation phase to eliminate persistent or intermittently reactivating bacilli, highlighting the need for additional scaffolds with activity against non-replicating mycobacterial populations [8]. These limitations emphasize the need for additional antimycobacterial scaffolds capable of acting against both replicating and non-replicating mycobacterial populations.

Recently, marine-derived fungi have attracted considerable attention as a potential source of bioactive natural products, including antibacterial, antifungal, and anticancer agents [9]. *Aspergillus* is one of the most abundant fungi in marine environments and has garnered significant interest due to its prolific ability to biosynthesize a vast array of structurally unique secondary metabolites with potent biological activities, including antimycobacterial activity [10]. However, most antimycobacterial studies of marine-derived fungal metabolites have focused on actively replicating mycobacteria, whereas compounds targeting non-replicating or dormancy-associated phenotypes remain less explored [11]. Despite the growing number of antimicrobial metabolites reported from marine-derived *Aspergillus* spp., investigations targeting dormant mycobacteria are still limited, and only a small number of compounds, including viomellein and xanthomegnin, have been tested under dormancy conditions [11,12,13]. This gap highlights marine-derived *Aspergillus* spp. as a promising source of metabolites for identifying scaffolds active against both replicating and non-replicating mycobacteria.

Building on our previous finding that fermentation extracts of the Indonesian marine-derived fungi *Aspergillus ostianus* and *A. flavus* displayed activity against both active and hypoxia-induced non-replicating *M. smegmatis* [13], we now isolated the responsible metabolites specifically from Indonesian marine-derived *A. ostianus* and investigated a Fe^3+^-reversible, iron-availability-dependent mechanism of activity. *M. smegmatis* is a non-pathogenic, fast-growing *Mycobacterium* species widely used as a surrogate model for *M. tuberculosis* research, thereby allowing safe and convenient in vitro evaluation of compounds against both replicating and non-replicating mycobacterial populations [14]. Structural investigation of the isolated metabolites revealed a hydroxamate moiety, a functional group associated with iron chelation, suggesting that these compounds might exert antimycobacterial effects by restricting iron availability, an essential requirement for mycobacterial growth [15,16,17]. Based on this rationale, we hypothesized that the hydroxamate functionality of these compounds restricts bioavailable iron for *M. smegmatis*, thereby inhibiting growth and viability. To test this hypothesis, we combined MIC determination, time-kill kinetics, scanning electron microscopy, release of UV-absorbing cytoplasmic material, and Fe^3+^-rescue assays under both aerobic and hypoxic atmospheres. We further examined whether activity was oxygen-tension-dependent, thereby addressing the central question of whether Fe^3+^ availability modulates activity against both replicating and hypoxia-adapted non-replicating mycobacterial states.

## 2. Results

### 2.1. Isolation and Identification of Active Compounds

**Figure 1:**
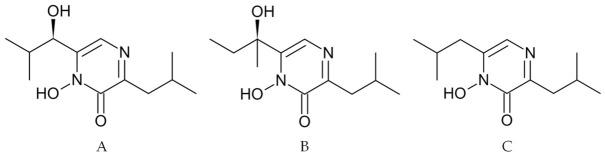
The chemical structures of neohydroxyaspergillic acid (**A**), hydroxyaspergillic acid (**B**), and neoaspergillic acid (**C**).

In our previous study [13] the ethyl acetate extracts of the biomass and fermentation medium of Indonesian marine-derived *Aspergillus ostianus* showed antimycobacterial activity against both active and hypoxia-induced non-replicating *M. smegmatis*. Bioassay-guided fractionation of these extracts (Figure S1, Supplementary Materials) afforded three active metabolites. The structures of compounds **1**–**3** were assigned by integrated analysis of MALDI-TOF-MS, ^1^H NMR, ^13^C NMR, HSQC, COSY, and HMBC data, followed by comparison with published spectroscopic data (Table 1 and Tables S1–S3 and Figures S2–S22, Supplementary Materials). Compound **1** was assigned as neohydroxyaspergillic acid (NHAA), compound **2** as hydroxyaspergillic acid (HAA), and compound **3** as neoaspergillic acid (NAA) (Figure 1). These compounds belong to the aspergillic-acid family of 2-hydroxypyrazine 1-*N*-oxides bearing a hydroxamate pharmacophore [18,19].

The assignment of compound **2** as hydroxyaspergillic acid was based primarily on the present 2D NMR correlations. COSY correlations established the aliphatic spin systems, while key HMBC correlations from H-5 and the side-chain protons to the pyrazinone quaternary carbons supported the attachment of the oxygenated tertiary side chain and the isobutyl side chain to the pyrazinone core (Figure S15, Supplementary Materials). The ^1^H and ^13^C NMR data showed some differences from the values reported by Guo et al.; however, the comparison data were recorded in CDCl_3_ at lower NMR frequencies, whereas the present data were acquired in DMSO-*d*_6_ at 600 MHz for ^1^H NMR and 150 MHz for ^13^C NMR (Table S2, Supplementary Materials). Therefore, the observed deviations are consistent with solvent- and condition-dependent chemical-shift differences in a hydroxamate/pyrazinone system and do not alter the planar structural assignment. The planar structure of compound **2** was assigned as hydroxyaspergillic acid.

Compound **3** was assigned as neoaspergillic acid based on MS and 1D/2D NMR correlations. COSY correlations established two isobutyl spin systems, and HMBC correlations connected these side chains to the pyrazinone core (Figure S22, Supplementary Materials). The pyrazinone core signals and the methylene/methine signals of the two isobutyl substituents agreed closely with the reported data of Zheng et al. [18]. The main discrepancy was restricted to the aliphatic methyl carbon region: the present HSQC data correlated the methyl proton signals at δH 0.87–0.88 with methyl carbons at δC 22.1 and 22.5, whereas Zheng et al. [18] reported the corresponding methyl carbons at approximately δC 19.4–19.5 (Table S3, Supplementary Materials). Because the methyl carbon assignments were directly supported by the present HSQC/HMBC data, they were retained as δC 22.1 and 22.5. This localized methyl-carbon difference does not affect the connectivity assignment of compound **3** as neoaspergillic acid.

Although NHAA, HAA, and NAA have been reported previously from terrestrial and marine fungal strains, and their antibacterial activity against replicating bacteria is known, we are not aware of previous reports demonstrating their bactericidal activity against hypoxia-induced non-replicating mycobacteria or the Fe^3+^-reversibility of this activity under oxygen-limited conditions.

### 2.2. Antimycobacterial Activity of NHAA, HAA, and NAA

The microdilution assay showed that all three compounds inhibited both aerobic and hypoxia-induced non-replicating *M. smegmatis* (Table 2). NHAA displayed the greatest potency, inhibiting growth at 1.56 µg/mL in both aerobic and hypoxic atmospheres. HAA and NAA followed with identical MIC values of 3.13 µg/mL, likewise unaffected by oxygen tension. On the other hand, the reference drug isoniazid (INH) retained an MIC of 3.13 µg/mL under aerobic conditions but required 25 µg/mL to achieve the same endpoint when oxygen was limited, representing an eight-fold loss of activity.

The aerobic INH MIC observed in the present study, 3.13 µg/mL, is comparable to published values for *M. smegmatis* under related experimental conditions. Elitas et al. reported an INH MIC of 3.13 µg/mL for wild-type *M. smegmatis*, while Sumii et al. and Kamiya et al. reported INH MIC values of 2.5 µg/mL under aerobic conditions and 25 µg/mL under hypoxic conditions against *M. smegmatis* [12,20,21,22]. This pattern is consistent with the present study, in which INH shifted from 3.13 µg/mL under aerobic conditions to 25 µg/mL under hypoxia. Because INH susceptibility in *M. smegmatis* varies with strain, medium composition, inoculum density, incubation time, oxygen tension, and endpoint readout, INH was used primarily as an internal assay-performance and oxygen-state comparator rather than as a universal benchmark.

### 2.3. Time Kill Assay Under Aerobic and Hypoxic Conditions

**Figure 2:**
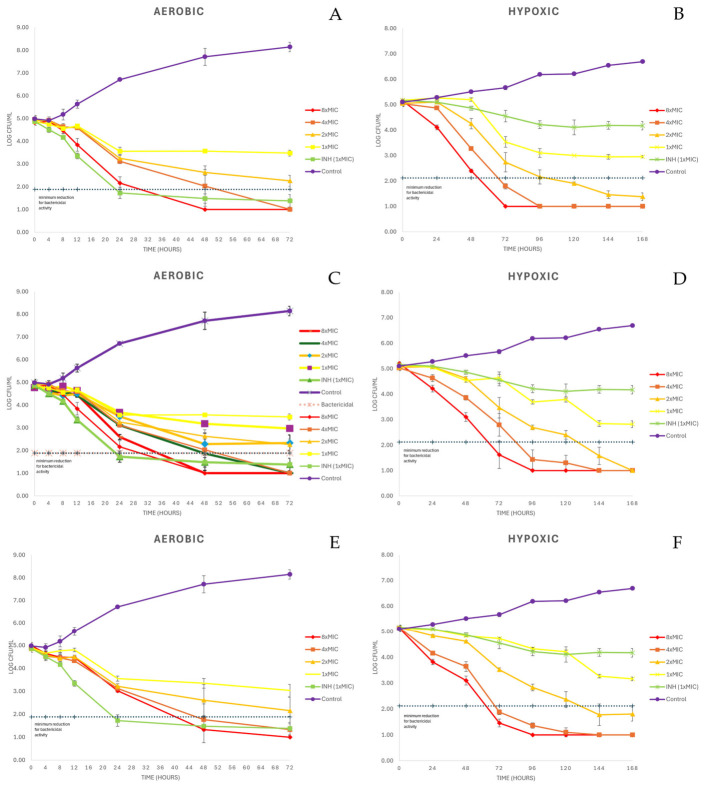
Time-kill kinetics of NHAA (**A**,**B**), HAA (**C**,**D**), and NAA (**E**,**F**) against aerobic and hypoxia-induced non-replicating *M. smegmatis*. Data are presented as mean ± SD from three independent experiments. INH was used at the MIC corresponding to each oxygen condition. A ≥ 3-log10 CFU reduction relative to the initial inoculum was considered bactericidal.

Figure 2 shows the time-kill kinetics of NHAA, HAA, and NAA compared with INH against *M. smegmatis* under aerobic and hypoxic atmospheres. Under oxygen-replete conditions, each pyrazinone produced a concentration-dependent decline in viable counts. NHAA drove the population below the bactericidal threshold of a 3-log10 reduction between 24–48 h when administered at 8× MIC and between 48–72 h at 4× MIC; whereas at 2× and 1× MIC it did not reach the bactericidal threshold and produced only modest reductions in CFU. HAA and NAA followed an almost identical profile. Both compounds eliminated more than 99.9% of bacilli between 24–48 h at 8× MIC and 4× MIC, while at 1–2× MIC they remained below the bactericidal cut-off and largely bacteriostatic. By contrast, INH at 3.13 µg/mL displayed bactericidal activity within 24 h against the aerobic culture. A ≥ 3-log_10_ CFU reduction relative to the initial inoculum, corresponding to approximately 99.9% killing, was considered bactericidal according to standard time-kill interpretation criteria [23].

Under hypoxia, untreated cultures showed a near-flat CFU trajectory, consistent with a hypoxia-adapted non-replicating phenotype. The eight-fold isoniazid MIC shift, from 3.13 to 25 µg/mL, further supported reduced INH susceptibility under this condition. INH did not produce bactericidal killing in the hypoxic time-kill assay, whereas NHAA, HAA, and NAA retained concentration-dependent bactericidal activity, although kill onset was modestly delayed. NHAA at 4× and 8× MIC produced a ≥3-log10 CFU reduction after 48–72 h, while 2× MIC crossed the bactericidal threshold only toward the end of the experiment. HAA showed a slightly slower profile, reaching bactericidal levels at 8× MIC within 48–72 h, at 4× MIC by 72–96 h, and at 2× MIC by 120–144 h. NAA mirrored NHAA at higher concentrations but required 120–144 h to achieve comparable killing at 2× MIC. Importantly, none of the three pyrazinones showed regrowth up to the final sampling point, supporting durable activity against non-replicating, INH-tolerant bacilli.

### 2.4. Scanning Electron Microscopy Analysis

**Figure 3:**
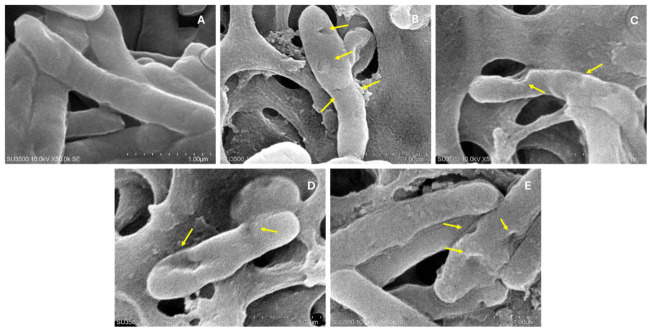
SEM images of *Mycobacterium smegmatis* after exposure to DMSO vehicle control (**A**), isoniazid (**B**), NHAA (**C**), HAA (**D**), and NAA (**E**) under aerobic conditions. Treatments were applied at 1× MIC for 48 h. Yellow arrows indicate representative morphological alterations. All figures were obtained at 10 kV and 50,000× magnification. Scale bar = 1 µm.

**Figure 4:**
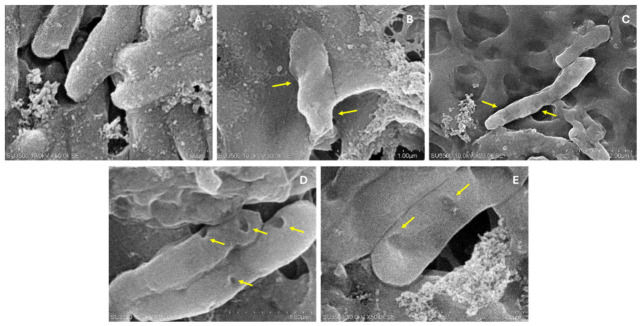
SEM images of *Mycobacterium smegmatis* after exposure to DMSO vehicle control (**A**), isoniazid (**B**), NHAA (**C**), HAA (**D**), and NAA (**E**) under hypoxic conditions. Treatments were applied at 1× MIC for 72 h. Yellow arrows indicate representative morphological alterations. All figures were obtained at 10 kV and 50,000× magnification Scale bar = 1 µm.

Scanning electron micrographs obtained at 10 kV and 50,000× magnification showed that exposure to the pyrazinone derivatives was associated with pronounced ultrastructural alterations of *M. smegmatis*, whereas untreated controls retained intact morphology (Figure 3 and Figure 4). In the DMSO-vehicle preparations under both aerobic and hypoxic atmospheres, bacilli displayed characteristic smooth, rod-shaped contours with continuous envelopes, indicating that the sample-preparation workflow did not itself compromise cell integrity.

Isoniazid, assayed at the MIC corresponding to each oxygen condition, produced less extensive surface alteration than the pyrazinones. Under aerobic conditions, INH-treated bacilli showed shallow longitudinal fissures and localized surface roughening. Under hypoxia, similar but less pronounced corrugation was observed, consistent with the reduced activity of INH against the non-replicating phenotype. Clear collapse or extensive surface defects were not observed in INH-treated hypoxic cells, supporting a lower degree of envelope damage than that observed after pyrazinone treatment. 

All three test compounds produced more pronounced surface alterations than INH. NHAA induced distinct invaginations, cavitation-like defects, and blister-like protrusions scattered along the cell surface, with several bacilli displaying deep surface pitting. HAA caused extensive membrane blebbing and local polar swelling, frequently accompanied by longitudinal cracks along the cell body. NAA showed a mixed pattern, combining surface craters, transverse fractures, and apparent loss of turgor. These deformities were observed in both aerobic and hypoxic specimens, suggesting that pyrazinone-associated envelope damage is not strongly dependent on oxygen tension and is consistent with the oxygen-independent MIC profile reported above.

Under hypoxic conditions (Figure 4), control bacilli displayed a denser extracellular matrix and a slightly wrinkled surface, whereas pyrazinone-treated cells showed marked envelope disruption, including deep fissures, membrane peeling, and large surface defects. These observations indicate that the compounds can damage the mycobacterial envelope even under oxygen-limited conditions. In contrast, comparable lesions were not observed in INH-exposed hypoxic cultures, consistent with the reduced activity of INH against non-replicating mycobacteria [24].

### 2.5. Intracellular Leakage Assay

**Figure 5:**
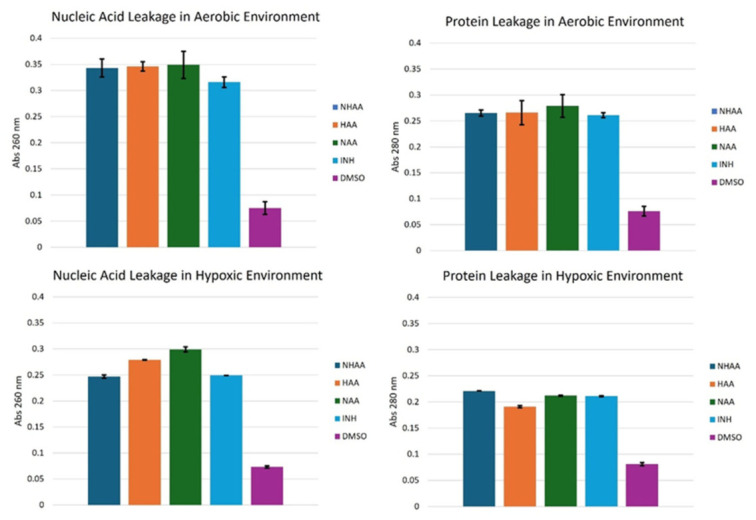
Release of UV-absorbing intracellular material from *M. smegmatis* after treatment with DMSO, INH, NHAA, HAA, or NAA at 1× MIC under aerobic and hypoxic conditions. Absorbance was measured at 260 nm and 280 nm after subtraction of compound-only blanks. Data are presented as mean ± SD from three independent experiments (*n* = 3).

The intracellular leakage assay further supported disruption of the *M. smegmatis* cell envelope by the three pyrazinone derivatives (Figure 5). Under aerobic conditions, exposure to NHAA, HAA, and NAA increased absorbance at 260 nm and 280 nm relative to the DMSO vehicle control, indicating release of UV-absorbing intracellular material. The OD_260_ signal is consistent with nucleic-acid-enriched material, whereas the OD_280_ signal is consistent with release of protein-enriched or aromatic biomolecules. Under hypoxia, overall absorbance values were lower than under aerobic conditions, but each pyrazinone still produced values approximately three-fold higher than the solvent control. NAA gave highest OD_260_ signal under oxygen limitation, whereas NHAA gave the highest OD_280_ signal, suggesting possible differences in the extent or composition of released intracellular material. Taken together, the leakage assay performed at 1× MIC indicates that MIC-level exposure to each pyrazinone compromises envelope integrity in both aerobic and hypoxic cultures. Agreement between the leakage readouts and SEM observations supports a mechanistic model in which these fungal metabolites perturb the mycobacterial envelope while retaining activity under oxygen-limited conditions.

### 2.6. Fe^3+^ Rescue Supports Iron-Dependent Antimycobacterial Activity

**Figure 6:**
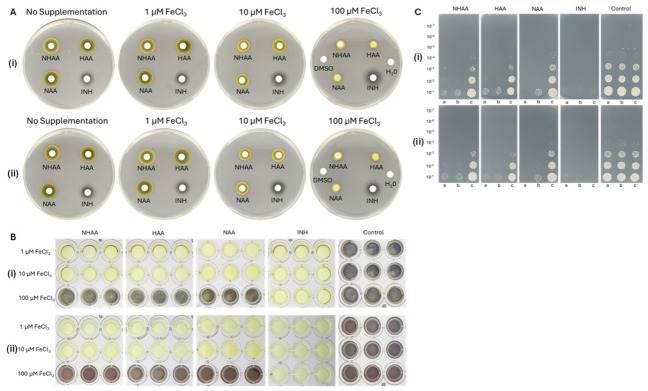
Effect of FeCl_3_ supplementation on the antimycobacterial activity of NHAA, HAA, NAA, and INH. (**A**) Agar-diffusion assay under (i) aerobic and (ii) hypoxic conditions. (**B**) MTT rescue assay under (i) aerobic and (ii) hypoxic conditions. Yellow wells indicate absence of visible formazan formation, whereas purple wells indicate formazan production by metabolically active bacilli. (**C**) Spot assay under (i) aerobic and (ii) hypoxic conditions with FeCl_3_ supplementation at (a) 1 µM, (b) 10 µM, and (c) 100 µM. Images are representative of three independent experiments.

Iron supplementation experiments were performed to test whether exogenous ferric ions could reverse the antimycobacterial activity of NHAA, HAA, and NAA (Figure 6). In agar-diffusion assays on Middlebrook 7H10, all three compounds produced clear inhibition zones in the absence of added iron. Zone diameters decreased with increasing FeCl_3_ supplementation and were abolished at 100 µM FeCl_3_, leaving only residual discoloration attributable to the pale-yellow pigment of the test compounds at the disc position. Isoniazid served as an iron-independent comparator; its inhibition zone was unchanged at every ferric concentration tested, indicating that the progressive loss of activity was specific to the pyrazinones. The same concentration-dependent rescue pattern was observed under hypoxia, suggesting that iron-dependent pyrazinone activity is preserved in the non-replicating state.

The microtiter MTT rescue assay provided a complementary metabolic readout. Wells containing low FeCl_3_ concentrations (1–10 µM) retained the pale-yellow color of unreduced MTT, consistent with loss of visible reductive metabolism, whereas wells supplemented with 100 µM FeCl_3_ turned dark purple, indicating restoration of formazan production comparable to the untreated growth control. This color reversion occurred for all three pyrazinones under both aerobic and hypoxic atmospheres but was absent in the isoniazid series, arguing against a nonspecific effect of FeCl_3_ on the assay readout. Viable-count spot assays reinforced these observations. Without iron supplementation, each compound markedly reduced culturable growth, whereas serial dilution spots re-emerged after supplementation with 100 µM FeCl_3_. Intermediate ferric levels produced partial rescue, supporting a graded relationship between extracellular Fe^3+^ availability and pyrazinone activity.

## 3. Discussion

The three hydroxamate pyrazinones NHAA, HAA, and NAA showed antimycobacterial activity against *M. smegmatis* in both actively replicating and hypoxia-induced non-replicating states, with MICs of 1.56–3.13 µg/mL (6.5–13 µM). This oxygen-tension-independent MIC profile is relevant because non-replicating bacilli are characteristically less susceptible to several first-line antitubercular drugs. Scanning electron microscopy and intracellular-leakage assays showed envelope alterations and release of UV-absorbing intracellular material after treatment, consistent with compromised membrane-envelope integrity. In addition, activity was reversed by exogenous Fe^3+^ in a concentration-dependent manner across three complementary readouts, including agar diffusion, MTT metabolic assay, and viable-count spot assay, whereas isoniazid activity was unaffected in the same rescue assays. These findings provide functional evidence that pyrazinone activity is Fe^3+^-reversible and iron-dependent in this model. However, because intracellular iron pools and compound–iron complexes were not directly measured, the data should be interpreted as consistent with iron sequestration or iron deprivation as a plausible mechanism, rather than as definitive proof of intracellular iron chelation.

Iron is a critical micronutrient for *M. tuberculosis* survival, and interference with iron acquisition is an emerging antitubercular strategy [17,25]. For example, Dragset et al. reported that a synthetic pyrazolopyrimidinone compound (PZP) inhibits mycobacterial growth by chelating intracellular Fe^3+^, effectively starving *M. tuberculosis* from within [17]. The aspergillic-acid family, to which NHAA, HAA, and NAA belong, likewise contains a hydroxamate functionality capable of forming metal complexes: Lebar et al. demonstrated that aspergillic acid binds iron to form ferriaspergillin, whereas Guo et al. characterized trichoderlate A as an Fe complex and trichoderlate B as an Al complex of hydroxyaspergillic acid using spectroscopic, HRMS, optical-rotation, and chemical methods [19,26]. By analogy, the hydroxamate pyrazinones isolated here may reduce Fe^3+^ availability to *M. smegmatis* through ferric complexation. However, because hydroxamate pyrazinones are known to form metal complexes, exogenous Fe^3+^ rescue could reflect restoration of bioavailable iron, extracellular complexation that reduces the free active pyrazinone, or both. The present assays do not distinguish extracellular Fe^3+^ complexation from intracellular iron chelation.

Compared with standard anti-TB drugs in this experimental model, the three pyrazinones displayed a distinct activity profile. Front-line drugs such as isoniazid (INH) are highly effective against rapidly dividing bacilli but are largely inactive on nonreplicating, dormant cells [27]. For instance, Karakousis et al. reported that INH’s transcriptional signature is abolished in dormant *M. tuberculosis*, reflecting its failure to kill nonreplicating bacilli [27]. In contrast, NHAA, HAA, and NAA retained activity under both aerobic and hypoxia-induced non-replicating conditions, suggesting that they target a vulnerability shared by replicating and hypoxia-adapted non-replicating mycobacteria. Similarly, naturally derived compounds viomellein and xanthomegnin were previously identified as anti-dormant *Mycobacterium* agents [12]; viomellein in particular was more active against dormant *M. bovis* BCG than growing cells, but it exhibited only weak activity against *M. smegmatis* [12]. Another example is nybomycin, a pyrrolo-pyridone antibiotic from a marine-derived *Streptomyces* that exhibits similar MICs (around 1 µg/mL) against both aerobic and hypoxic *M. smegmatis*. Nybomycin kills mycobacteria by targeting DNA topoisomerase function with concomitant morphological changes, whereas NHAA, HAA, and NAA showed Fe^3+^-reversible activity consistent with an iron-availability-dependent mode of action [28]. Thus, the dual-condition activity of NHAA, HAA, and NAA, together with Fe^3+^ reversibility, supports a mechanistically distinct activity profile relative to INH and previously reported anti-dormant natural products such as viomellein, xanthomegnin, and nybomycin, although direct intracellular iron chelation remains to be demonstrated.

Although the aspergillic-acid pyrazinone scaffold has been known for decades and its class-level iron-binding chemistry is established, the present study provides a new biological context and a testable mechanistic hypothesis for NHAA, HAA, and NAA. To our knowledge, this is the first demonstration that these compounds retain low-micromolar activity and bactericidal effects against hypoxia-induced non-replicating *M. smegmatis* and that this activity is Fe^3+^-reversible under both aerobic and hypoxic conditions. The contribution of this work therefore lies not in new structural discovery, but in linking a known fungal chelator class to Fe^3+^-reversible killing of a hypoxia-adapted non-replicating mycobacterial phenotype.

This study has several limitations that should be acknowledged. Antimycobacterial activity was characterized in *M. smegmatis*, a BSL-1 mycobacterial model suitable for preliminary mechanism-of-action studies; therefore, extension to virulent *M. tuberculosis* and additional dormancy models remains an important next step. Hypoxia was selected because oxygen limitation is a well-established trigger of non-replicating persistence in mycobacteria and because the resulting phenotype can be functionally monitored by reduced INH susceptibility [29,30,31,32]. However, hypoxia alone does not reproduce all dormancy-associated stresses encountered in vivo. Nutrient starvation and oxidative/nitrosative stress models are complementary systems, and future studies should determine whether the Fe^3+^-reversible activity observed here extends to those additional dormancy-associated states. Sub-MIC time-kill concentrations, such as ½× and ¼× MIC, were not included because limited quantities of purified natural products remained after completion of structural and biological assays. Therefore, the present time-kill data define bactericidal activity at and above the MIC, but do not fully describe sub-inhibitory pharmacodynamic effects. Future studies with re-isolated or synthetically prepared material should include ¼× and ½× MIC exposures to define sub-MIC growth effects and recovery kinetics. The mechanistic interpretation advanced here is based on functional Fe^3+^ rescue rather than direct demonstration of intracellular iron chelation. We did not directly quantify intracellular labile iron, determine Fe^3+^-binding affinity or stoichiometry for each congener, or detect compound–Fe complexes inside mycobacterial cells. Therefore, the Fe^3+^ rescue data support Fe^3+^-reversible and iron-dependent antimycobacterial activity, but they do not by themselves prove intracellular iron chelation. The hydroxamate-containing 2-hydroxypyrazine-1-*N*-oxide scaffold has established class-level ferric-complex chemistry, and the biological hypothesis tested here was whether Fe^3+^ availability modulates antimycobacterial activity in living cultures. The convergence of agar-diffusion, MTT, and viable-count rescue assays under both aerobic and hypoxic conditions supports the biological relevance of Fe^3+^ availability, while direct UV–Vis titration, isothermal titration calorimetry, LC–MS detection of compound–Fe complexes, intracellular labile-iron assays, ICP-MS, or metal-specificity profiling would be required to define the precise binding events and cellular site of action.

Host–cell selectivity assessment, structure–activity interrogation of the 2-hydroxypyrazine-1-*N*-oxide scaffold, metal-specificity profiling, pharmacokinetic characterization, intracellular iron measurements, and resistance-emergence studies are necessary follow-up studies to test and refine this Fe^3+^-reversible, iron-dependent activity model.

## 4. Materials and Methods

### 4.1. Fungus and Extract Preparation

The marine-derived fungal strain *Aspergillus ostianus* was isolated from seagrass roots collected at Sekrikil Beach, Lamongan, East Java, Indonesia [13]. The fungus was cultured at 25 ± 2 °C on YPD agar prepared in artificial seawater (ASW), consisting of yeast extract (10 g/L) (Oxoid, Basingstoke, UK), peptone (20 g/L) (Oxoid, Basingstoke, UK), dextrose (20 g/L) (Oxoid, Basingstoke, UK), agar (15 g/L) (Oxoid, Basingstoke, UK), NaCl (24.6 g/L) (Merck, Darmstadt, Germany), KCl (0.67 g/L) (Merck, Darmstadt, Germany), CaCl_2_·2H_2_O (1.36 g/L) (Merck, Darmstadt, Germany), MgSO_4_·7H_2_O (6.29 g/L) (Merck, Darmstadt, Germany), MgCl_2_·6H_2_O (4.66 g/L) (Merck, Darmstadt, Germany), and NaHCO_3_ (0.18 g/L) (Merck, Darmstadt, Germany), with the final pH adjusted to 7.2–7.5 [33,34]. After sporulation was established, approximately after 5 days, ten agar fragments (1 × 1 cm) were cut using a sterile blade, transferred into ten 250 mL flasks containing 100 mL of YPD broth in ASW, and incubated at 25 ± 2 °C with agitation at 150 rpm. After pellets were formed, all pellets were transferred to ten 2 L flasks containing 400 mL YPD broth in ASW and incubated at 25 ± 2 °C in static condition for up to 21 days [34]. After incubation, the biomass and the fermentation medium were separated by filtration using Büchner funnel. All fermentation products were extracted using ethyl acetate as solvent. The biomass was extracted by maceration for 3 × 24 h using a biomass-to-solvent ratio of 1:10, with solvent replacement every 24 h. The fermentation medium was extracted three times by liquid–liquid extraction using a 1:1 medium-to-solvent ratio. The obtained extracts (biomass extract (BEA) and media extract (MEA)) were concentrated under vacuum at 40 °C until dry extracts were obtained. Dried extracts were stored at −20 °C until further usage [13].

### 4.2. Antimycobacterial Investigation Using Microdilution and MTT Assay

This assay was used to evaluate activity against aerobic and hypoxia-induced non-replicating *M. smegmatis*. The *M. smegmatis* strain used in this study was *M. smegmatis* mc^2^155, obtained from the Laboratory of Natural Products for Drug Discovery, Graduate School of Pharmaceutical Sciences, The University of Osaka [12,14]. First, *M. smegmatis* was cultured in Middlebrook 7H9 (Nacalai Tesque, Inc., Kyoto, Japan) medium supplemented with NaCl at a final concentration of 0.04% (*w*/*v*), glucose at 0.2% (*w*/*v*), and Tween 80 at 0.05% (*v*/*v*), and incubated at 37 °C and 150 rpm for 24 h. The OD_600_ was then measured, and the suspension was adjusted to a cell density of 10^5^ CFU/mL [12,13,28].

For aerobic assay, a total of 196 μL bacterial suspension was added to the first column of a 96-well plate, followed by 100 μL in subsequent columns. Four microliters of sample (10 mg/mL) were added to the first column, mixed, and serially diluted (1:2) across the plate. Isoniazid and DMSO served as positive and negative controls, respectively. Plates were incubated at 37 °C for 24 h, followed by addition of 10 μL MTT reagent (5 mg/mL) and further incubation for 12 h. Afterward, 50 μL of solubilizing agent (1:1 SDS 20% and DMF 50% in 2% acetic acid) was added, mixed, and the plate was incubated at 25 ± 2 °C for 3 h. MIC assays were performed in three independent biological experiments, each with technical triplicates. The MIC was defined as the lowest concentration showing no visible MTT reduction and no measurable increase in OD_560_ relative to the blank-corrected control [12,13,22]. When replicate endpoints differed by one two-fold dilution, the modal MIC was reported; if no single modal value occurred, the median two-fold dilution endpoint was reported. All compounds were prepared as DMSO stock solutions. In the MIC assay, the first dilution well contained 4 µL of compound stock in a final volume of 200 µL, corresponding to a maximum DMSO concentration of 2% (*v*/*v*), followed by two-fold serial dilution. A parallel DMSO-only dilution series was included as the vehicle control. For time-kill, SEM, intracellular leakage, and Fe^3+^-rescue assays, DMSO vehicle controls were matched to the DMSO concentration present in the corresponding compound-treated samples.

For hypoxic assays (dormant state), the bacterial suspension was prepared as described above. When the OD_600_ reached 0.5, cultures were placed in an anaerobic jar purged with nitrogen to maintain oxygen levels below 0.2%, and then incubated at 37 °C with agitation at 150 rpm for 7 days [12,13]. Oxygen concentration was monitored daily and adjusted with nitrogen as needed. The microdilution assay followed the same procedure as under aerobic conditions, except plates were incubated anaerobically at 37 °C for 3 days [35]. Afterwards, 10 μL of MTT reagent was added and plates were further incubated anaerobically for 12 h, followed by addition of solubilising agent, gentle mixing and 3 h incubation at 25 ± 2 °C before OD_560_ measurement. The non-replicating-persistence protocol described above is the established platform used in multiple peer-reviewed studies at the Laboratory of Natural Products for Drug Discovery (The University of Osaka) and has been used in a series of peer-reviewed studies of marine-derived antimycobacterial compounds, including trichoderins, nybomycin and viomellein/xanthomegnin [12,22,28,36]. Within this established framework, the ≥8-fold rise in the isoniazid MIC (from 3.13 to 25 µg/mL) between aerobic and hypoxic conditions was used as a functional indicator that the cultures had acquired a hypoxia-adapted, INH-tolerant non-replicating phenotype. Test compounds that maintained their MIC under hypoxia were therefore interpreted as active against this operationally defined NRP phenotype [12,28,36].

### 4.3. Bioassay-Guided Isolation of the Active Compounds

BEA and MEA were partitioned by liquid–liquid extraction using *n*-hexane and 90% aqueous MeOH at a 1:1 ratio, following solvent-partitioning workflows commonly used to simplify crude natural-product extracts prior to chromatographic purification of bioactive secondary metabolites from marine-derived fungi [34,37]. Based on bioassay-guided fractionation, the 90% MeOH-soluble portion of MEA, with an MIC of 6.25 µg/mL against active and hypoxia-induced non-replicating *M. smegmatis*, was fractionated using flash column chromatography with ODS Cosmosil 75C18-OPN (Nacalai Tesque, Inc., Kyoto, Japan) as the stationary phase and a gradient of 30–100% MeOH, resulting in 15 fractions (Fr. M1–Fr. M15). Among these fractions, Fr. M7 showed strong antimycobacterial activity and was available in sufficient amount for further purification, with MIC values of 3.13 µg/mL against active and 6.25 µg/mL against hypoxia-induced non-replicating *M. smegmatis*. This fraction was further separated by reverse-phase HPLC [ODS C18-ARII (10 mm i.d. × 250 mm) (Hitachi High-Tech Science Corporation, Tokyo, Japan), eluted with 60% MeOH containing 0.1% trifluoroacetic acid (TFA) at a flow rate of 2 mL/min, with UV detection at 210 nm] to obtain three subfractions, SFr. M7-1–SFr. M7-3. Based on activity, SFr. M7-2 showed potent activity, with MIC values of 3.135 µg/mL against active and 6.25 µg/mL against hypoxia-induced non-replicating *M. smegmatis*. Further subfractionation of SFr. M7-2 was performed by reverse-phase HPLC using the previous system but eluted with 50% MeOH containing 0.1% TFA to obtain five subfractions (SFr. M7-2-1–SFr. M7-2-5). Among these, SFr. M7-2-4 showed the most potent activity, with MIC values of 1.56 µg/mL against active and 3.13 µg/mL against hypoxia-induced non-replicating *M. smegmatis*. Further purification of this subfraction was performed by reverse-phase HPLC [ODS C18-ARII (4.6 mm i.d. × 250 mm), eluted with 47.5% MeOH containing 0.1% TFA at a flow rate of 0.8 mL/min, with UV detection at 210 nm] to obtain compound **1** [18].

Another subfractionation was performed for Fr. M6 by reverse-phase HPLC [ODS C18-ARII (10 mm i.d. × 250 mm), eluted with 57.5% MeOH containing 0.1% TFA at a flow rate of 2 mL/min, with UV detection at 210 nm] to obtain three subfractions (SFr. M6-1–SFr. M6-3). SFr. M6-1 showed potent activity, with an MIC of 1.56 µg/mL against both active and hypoxia-induced non-replicating *M. smegmatis*. Further purification of SFr. M6-1 was performed by reverse-phase HPLC using the same system but eluted with 50% MeOH containing 0.1% TFA to obtain compound **2** [19].

Further chemical investigation was performed on BEA. The 90% MeOH-soluble portion was fractionated using flash column chromatography with the same system as described above to obtain ten fractions (Fr. B1–Fr. B10). Among these, Fr. B6 was selected for further subfractionation because of its activity, with an MIC of 12.5 µg/mL against both active and hypoxia-induced non-replicating *M. smegmatis*. Fr. B6 was purified by reverse-phase HPLC [ODS C18-ARII (10 mm i.d. × 250 mm), eluted with 70% MeOH containing 0.1% TFA at a flow rate of 2 mL/min, with UV detection at 210 nm]. This process led to the isolation of compound **3**, with an MIC of 3.13 µg/mL against both active and hypoxia-induced non-replicating *M. smegmatis*. Compounds **1**–**3** were identified by MALDI-TOF-MS, recorded on a SpiralTOF™ JEOL JMS-S3000 mass spectrometer (JEOL Ltd., Tokyo, Japan), and 1D/2D NMR analysis using Varian Inova 600-II NMR spectrometer (Varian, Inc., Palo Alto, CA, USA), followed by comparison with published spectroscopic data [18,19].

### 4.4. Time-Kill Assay

Time-kill assays were performed to distinguish the bactericidal or bacteriostatic effects of compounds **1**–**3** against *M. smegmatis* under aerobic and hypoxia-induced non-replicating conditions, following standardized bactericidal/time-kill testing approaches [23,38,39]. A standardized inoculum of 10^5^ CFU/mL in 7H9 medium was treated with each compound at 1×, 2×, 4×, and 8× the MIC determined under the corresponding oxygen condition [39,40]. Samples were collected at designated time points up to 72 h for aerobic conditions and 168 h for hypoxic conditions, serially diluted, and plated on Middlebrook 7H10 agar supplemented with glycerol (5 mL/L). Plates were incubated at 37 °C for 48 h (aerobic) or 7 days (hypoxic), after which colonies were counted and CFUs plotted over time. DMSO was used as the vehicle control, and INH was used at the MIC corresponding to each condition as the reference control. A ≥ 3 log10 CFU reduction compared with initial cell counts was considered indicative of bactericidal activity [23]. All experiments were performed as three independent biological replicates, each in technical triplicate; data are presented as mean ± SD, and differences in CFU counts between treatments and the DMSO vehicle control at each sampling time were analysed by two-way ANOVA with Tukey’s HSD post hoc test (α = 0.05) [38].

### 4.5. Scanning Electron Microscope (SEM) Analysis

SEM sample preparation followed the protocol of Liu et al. with modifications [41]. *M. smegmatis* cultures were treated under aerobic (48 h) and hypoxic (72 h) conditions with each compound at 1× MIC, alongside 1× MIC isoniazid and DMSO as controls. The MICs used for the test compounds and INH followed the condition in which the assay was performed (aerobically or hypoxically). After incubation, cells were filtered onto 0.2 µm membranes (Sartorius), washed three times with sterile PBS (10 mM, pH 7.4), and fixed overnight at 4 °C with 2.5% glutaraldehyde. Membranes were then washed and dehydrated in a graded ethanol series (30% to 100%), with two final steps in absolute ethanol. Samples were air-dried, mounted on aluminium studs using carbon tape, and sputter-coated with gold for 3 min. Cellular ultrastructure was examined using a Hitachi SU3500 SEM (Research Center for Nanoscience and Nanotechnology/PPNN, ITB, Bandung, Indonesia) at 10 kV and 50,000× magnification [41,42].

### 4.6. Investigation of the Mycobacterial Intracellular Leakage

The *M. smegmatis* suspension was prepared as described above [12,22]. A bacterial suspension equivalent to 10^8^ CFU/mL was mixed with each compound to reach a final concentration of 1× MIC and incubated at 37 °C under aerobic conditions for 48 h or hypoxic conditions for 72 h, because MIC-level exposure is commonly used in intracellular leakage assays to evaluate antimicrobial-induced disruption of bacterial membrane or envelope integrity [43,44]. After incubation, the suspension was centrifuged at 6000 rpm for 20 min. The supernatant was collected and analyzed by UV–Vis spectrophotometry [44,45]. INH at 1× MIC and DMSO were used as controls, and each compound at the corresponding concentration without bacterial suspension was used as a blank. Release of nucleic-acid-enriched material was monitored at 260 nm, whereas release of A280-absorbing biomolecules was monitored at 280 nm [43,44,45]. A compound-in-medium-only blank was subtracted from all treated samples before reporting to control for intrinsic UV absorbance of the pyrazinone chromophores [43].

### 4.7. Fe^3+^ Rescue Assay to Assess Iron-Dependent Antimycobacterial Activity

Fe^3+^ rescue assays were performed to determine whether exogenous ferric iron attenuated the antimycobacterial activity of compounds **1**–**3**, following previous studies showing that iron availability and iron chelation can modulate mycobacterial growth and drug susceptibility [17,46]. The rationale for this assay was also supported by the established Fe^3+^-binding chemistry of hydroxamate pyrazinones, including aspergillic-acid/ferriaspergillin formation and metal complexes of hydroxyaspergillic acid [19,26].

To test whether exogenous Fe^3+^ could rescue the antimycobacterial activity of compounds **1**–**3**, iron-rescue assays were conducted using agar diffusion [38]. Solutions of compounds **1**–**3** were prepared at 10 mg/mL. Filter-sterilized FeCl_3_·6H_2_O solution was added to sterile molten 7H10 agar to final concentrations of 1, 10, and 100 µM [46]. Then, 100 µL of bacterial suspension (10^8^ CFU/mL) was mixed with 7H10 agar supplemented with glycerol (5 mL/L), poured into Petri dishes, and allowed to cool and solidify. Paper discs were placed on the agar surface, and 10 µL of each compound solution was applied to the discs. Water and DMSO were used as controls, and INH at 1 mg/mL was used as the reference drug. Plates were pre-incubated for 30 min at 25 ± 2 °C to allow diffusion, then incubated at 37 °C for 24 h. Zones of inhibition were compared between plates without FeCl_3_ supplementation and plates supplemented with 1–100 µM FeCl_3_ [46]. For hypoxic agar-diffusion assays, plates were prepared identically and incubated in an anaerobic jar under oxygen-limited conditions (<0.2% O_2_) at 37 °C for 3 days.

In addition to agar diffusion, the effect of Fe^3+^ supplementation on antimycobacterial activity was evaluated using MTT and drop-plate assays in microplates to provide complementary metabolic and viable-growth readouts [47]. Briefly, bacterial suspension (10^6^ CFU/mL) was added to each well, followed by compounds **1**–**3** at a final concentration of 1× MIC. FeCl_3_ was then added to final concentrations of 1, 10, and 100 µM [46]. Wells containing DMSO and FeCl_3_ without test compounds were included to assess iron toxicity, and INH at the MIC corresponding to each oxygen condition was used as the reference control. All assays were performed in triplicate. Microplates were incubated at 37 °C for 48 h under aerobic conditions or for up to 7 days under hypoxic conditions. For the drop-plate assay, 20 µL aliquots were serially diluted, and 5 µL aliquots from each dilution were spotted onto 7H10 agar without FeCl_3_ supplementation. Plates were incubated at 37 °C for 48 h to allow growth of surviving colonies [48,49]. For the MTT assay, 10 µL of MTT reagent (5 mg/mL) was added to each well and incubated for a further 12 h. Then, 50 µL of solubilizing agent was added, mixed, and incubated at 25 ± 2 °C for 3–4 h. Yellow wells indicated absence of visible MTT reduction, whereas purple wells indicated formazan formation by metabolically active cells [12]. A DMSO–FeCl_3_ control series at 1, 10, and 100 µM FeCl_3_ without compounds was included in each rescue-assay run to confirm that the ferric ion concentrations used were not inhibitory to *M. smegmatis*.

### 4.8. Statistical Analysis

In this study, statistical analyses were performed in IBM SPSS Statistics v29. One-way and two-way ANOVA with Tukey’s HSD post hoc test were used to evaluate between-group differences, as appropriate (α = 0.05). All results are presented as mean ± SD from three independent experiments performed in triplicate, unless stated otherwise.

## 5. Conclusions

In conclusion, we report the isolation of three hydroxamate pyrazinones—neohydroxyaspergillic acid (NHAA), hydroxyaspergillic acid (HAA), and neoaspergillic acid (NAA)—from Indonesian marine-derived *Aspergillus ostianus* and demonstrate that all three show low-micromolar antimycobacterial activity against both aerobic and hypoxia-induced non-replicating *M. smegmatis*. Concentration-dependent Fe^3+^ rescue, supported by scanning electron microscopy and release of UV-absorbing intracellular material, is consistent with iron-dependent activity and envelope disruption across replicative states. These findings establish a functional link between a known class of fungal hydroxamate pyrazinones and Fe^3+^-reversible killing of hypoxia-adapted non-replicating mycobacteria. The compounds therefore represent chemically tractable scaffolds for further investigation of nutrient-deprivation-based antimycobacterial strategies. Evaluation against virulent *M. tuberculosis* in Wayne and nutrient-starvation models, structure–activity interrogation of the 2-hydroxypyrazine-1-*N*-oxide scaffold, cytotoxicity/selectivity assessment, and combination testing with established antitubercular drugs are logical next steps to test and refine this Fe^3+^-reversible, iron-dependent activity model.

## Figures and Tables

**Table 1:** NMR spectral data of neohydroxyaspergillic acid, hydroxyaspergillic acid, and neoaspergillic acid (recorded in DMSO-*d*_6_; δ in ppm; *J* in Hz). The prime (′) and double prime (″) notations denote atomic assignments on the two respective aliphatic side chains attached to the main heterocyclic core shown in the structures.

Position	Compound 1 Neohydroxyaspergillic Acid		Compound 2 Hydroxyaspergillic Acid		Compound 3 Neoaspergillic Acid	
	δC	δH (*J* in Hz)	δC	δH (*J* in Hz)	δC	δH (*J* in Hz)
1						
2	152.0, qC		152.2, qC		152.4, qC	
3	154.4, qC		154.3, qC		154.5, qC	
4						
5	119.9, CH	7.26 (1H, s)	120.2, CH	7.42 (1H, s)	121.6, CH	7.13 (1H, s)
6	141.1, qC		142.7, qC		137.7, qC	
1′	69.72, CH	4.64 (1H, d, *J* = 4.2)	72.4, qC		36.2, CH_2_	2.51 (2H, d, *J* = 7.2)
2′	31.6, CH	2.03 (1H, m)	32.3, CH_2_	2.05 (1H, m)	26.3, CH	2.02 (1H, m)
				1.75 (1H, m)		
3′	19.6, CH_3_	0.94 (3H, d, *J* = 7.2)	26.8, CH_3_	1.53 (3H, s)	22.1, CH_3_	0.87 (3H, d, *J* = 4.2)
4′	15.9, CH_3_	0.77 (3H, d, *J* = 7.2)	8.2, CH_3_	0.69 (3H, t, *J* = 7.2)	22.1, CH_3_	0.87 (3H, d, *J* = 4.2)
1″	41.6, CH_2_	2.57 (2H, d, *J* = 7.0)	41.5, CH_2_	2.57 (2H, m)	41.5, CH_2_	2.56 (2H, d, *J* = 7.2)
2″	26.3, CH	2.13 (1H, m)	26.2, CH	2.13 (1H, m)	26.2, CH	2.12 (1H, m)
3″	22.5, CH_3_	0.87 (3H, d, *J* = 6.6)	22.5, CH_3_	0.88 (3H, d, *J* = 6.7)	22.5, CH_3_	0.88 (3H, d, *J* = 4.2)
4″	22.4, CH_3_	0.87 (3H, d, *J* = 6.6)	22.4, CH_3_	0.88 (3H, d, *J* = 6.7)	22.5, CH_3_	0.88 (3H, d, *J* = 4.2)

**Table 2:** Minimum inhibitory concentrations (MIC, µg/mL) of NHAA, HAA, NAA and isoniazid against *M. smegmatis* under aerobic (active) and hypoxic (non-replicating) conditions.

Compounds	MIC (µg/mL)	
	Aerobic	Hypoxic
Neohydroxyaspergillic acid (NHAA)	1.56	1.56
Hydroxyaspergillic acid (HAA)	3.13	3.13
Neoaspergillic acid (NAA)	3.13	3.13
Isoniazid	3.13	25

## Data Availability

The data supporting the findings of this study are available from the corresponding author upon reasonable request.

## Supplementary Materials

The following supporting information can be downloaded at: https://www.mdpi.com/article/10.3390/md24070236/s1, Figure S1: Antimycobacterial-activity-guided fractionation of the biomass (BEA) and fermentation-medium (MEA) ethyl acetate extracts of marine-derived *Aspergillus ostianus*; Figure S2: MALDI-TOF-MS and NMR spectra of compound **1**; Figure S3: ^1^H NMR spectrum of compound **1** in DMSO-*d6*; Figure S4: ^13^C NMR spectrum of compound **1** in DMSO-*d6*; Figure S5: HSQC spectrum of compound **1** in DMSO-*d6*; Figure S6: COSY spectrum of compound **1** in DMSO-*d6*; Figure S7: HMBC spectrum of compound **1** in DMSO-*d6*; Table S1: ^1^H NMR and ^13^C NMR comparison of compound **1** and Neohydroxyaspergillic acid (NHAA) [18]; Figure S8: COSY and HMBC correlations of compound **1** (neohydroxyaspergillic acid/NHAA); Figure S9: MALDI-TOF-MS and NMR spectra of compound **2**; Figure S10: ^1^H NMR spectrum of compound **2** in DMSO-*d6*; Figure S11: ^13^C NMR spectrum of compound **2** in DMSO-*d6*; Figure S12: HSQC spectrum of compound **2** in DMSO-*d6*; Figure S13: COSY spectrum of compound **2** in DMSO-*d6*; Figure S14: HMBC spectrum of compound **2** in DMSO-*d6*; Table S2: ^1^H NMR and ^13^C NMR comparison of compound **2** and Hydroxyaspergillic acid (HAA) [19]; Figure S15: COSY and HMBC correlations of compound **2** (hydroxyaspergillic acid/HAA); Figure S16: MALDI-TOF-MS and NMR spectra of compound **3**; Figure S17: ^1^H NMR spectrum of compound **3** in DMSO-*d6*; Figure S18: ^13^C NMR spectrum of compound **3** in DMSO-*d6*; Figure S19: HSQC spectrum of compound **3** in DMSO-*d6*; Figure S20: COSY spectrum of compound **3** in DMSO-*d6*; Figure S21: HMBC spectrum of compound **3** in DMSO-*d6*; Table S3: ^1^H NMR and ^13^C NMR comparison of compound **3** and Neoaspergillic acid (NAA) [18]; Figure S22: COSY and HMBC correlations of compound **3** (Neoaspergillic acid/NAA).
