# Supplementary material for: Iron-Reversible Bactericidal Activity of Marine-Derived Aspergillus ostianus Hydroxamate Pyrazinones Against Replicating and Hypoxia-Induced Non-Replicating Mycobacterium smegmatis
Source: Mar Drugs. 2026 Jul 3;24(7):236. doi: 10.3390/md24070236 (PMC13412789; doi:10.3390/md24070236)
Supplement: Supplementary file 1 [file marinedrugs-24-00236-s001.zip › marinedrugs-4328893-supplementary.pdf]

Supporting Information

Iron-Reversible Bactericidal Activity of Marine-Derived *Aspergillus ostianus* Hydroxamate Pyrazinones against Replicating and Hypoxia-Induced Non-Replicating *Mycobacterium smegmatis*

Muhammad Azhari[1], Shinnosuke Isshiki[2], Riku Horinouchi[2], Marlia Singgih[1], Masayoshi Arai[2], Afrillia Nuryanti Garmana[3], Rika Hartati[4], Yuni Elsa Hadisaputri[5], Nunung Yuniarti[6], and Elin Julianti[1]*

*** Correspondence: elin_julianti@itb.ac.id**

List of Supporting Information

Figure S1. Antimycobacterial-activity-guided fractionation of the biomass (BEA) and fermentation-medium (MEA) ethyl acetate extracts of marine-derived *Aspergillus ostianus* 3
Figure S2. MALDI-TOF-MS spectrum of compound **1** 4
Figure S3. ^{1}H NMR spectrum of compound **1** in DMSO-*d6* 5
Figure S4. ^{13}C NMR spectrum of compound **1** in DMSO-*d6* 6
Figure S5. HSQC spectrum of compound **1** in DMSO-*d6* 7
Figure S6. COSY spectrum of compound **1** in DMSO-*d6* 8
Figure S7. HMBC spectrum of compound **1** in DMSO-*d6* 9
Table S1. ^{1}H NMR and ^{13}C NMR comparison of compound **1** and Neohydroxyaspergillic acid (NHAA) (Zheng *et al.*, 2013) 10
Figure S8. COSY and HMBC correlations of compound **1** (neohydroxyaspergillic acid/NHAA). 11
Figure S9. MALDI-TOF-MS spectrum of compound **2** 12
Figure S10. ^{1}H NMR spectrum of compound **2** in DMSO-*d6* 13
Figure S11. ^{13}C NMR spectrum of compound **2** in DMSO-*d6* 14
Figure S12. HSQC spectrum of compound **2** in DMSO-*d6* 15
Figure S13. COSY spectrum of compound **2** in DMSO-*d6* 16
Figure S14. HMBC spectrum of compound **2** in DMSO-*d6* 17
Table S2. ^{1}H NMR and ^{13}C NMR comparison of compound **2** and Hydroxyaspergillic acid (HAA) (Guo *et al.*, 2019). 18
Figure S15. COSY and HMBC correlations of compound **2** (hydroxyaspergillic acid/HAA). 19
Figure S16. MALDI-TOF-MS spectrum of compound **3** 20
Figure S17. ^{1}H NMR spectrum of compound **3** in DMSO-*d6* 21

Figure S18. ^{13}C NMR spectrum of compound **3** in DMSO-*d6* 22
Figure S19. HSQC spectrum of compound **3** in DMSO-*d6* 23
Figure S20. COSY spectrum of compound **3** in DMSO-*d6* 24
Figure S21. HMBC spectrum of compound **3** in DMSO-*d6* 25
Table S3. ^{1}H NMR and ^{13}C NMR comparison of compound **3** and Neoaspergillic acid (NAA) (Zheng *et al*., 2013) 26
Figure S22. COSY and HMBC correlations of compound **3** (Neoaspergillic acid/NAA). 27

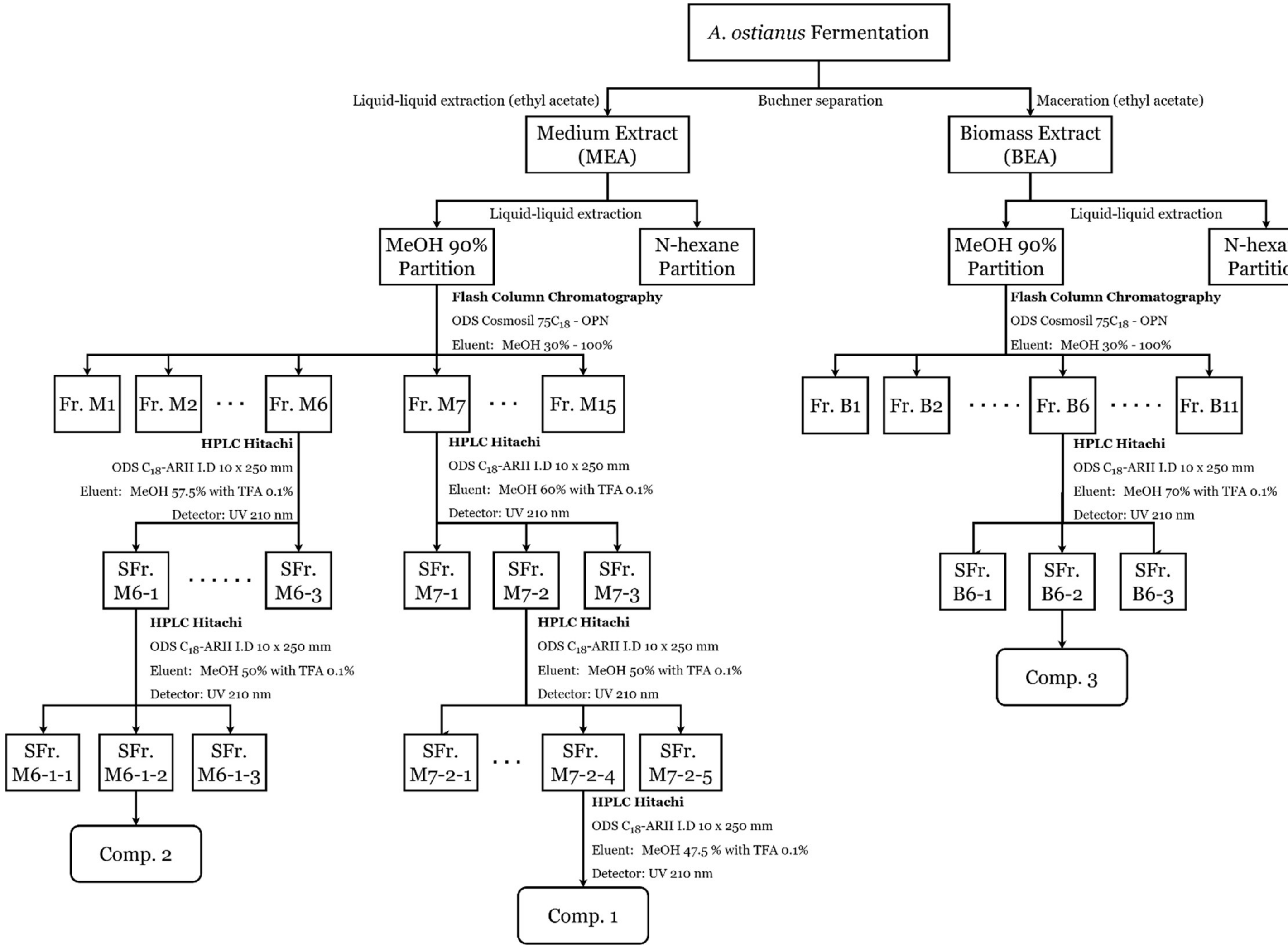

Figure S1. Antimycobacterial-activity-guided fractionation of the biomass (BEA) and fermentation-medium (MEA) ethyl acetate extracts of marine-derived *Aspergillus ostianus*

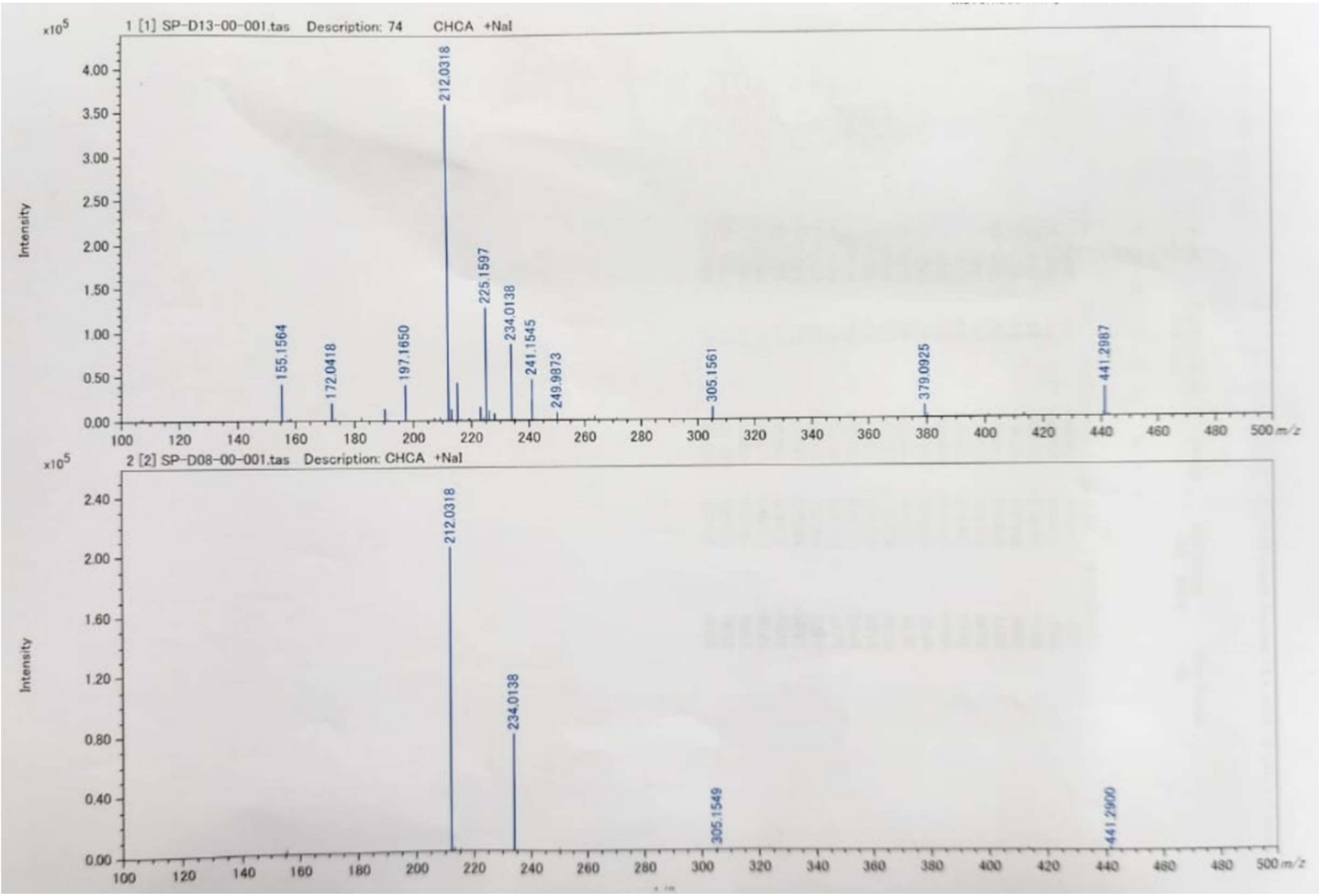

Figure S2. MALDI-TOF-MS spectrum of compound **1**

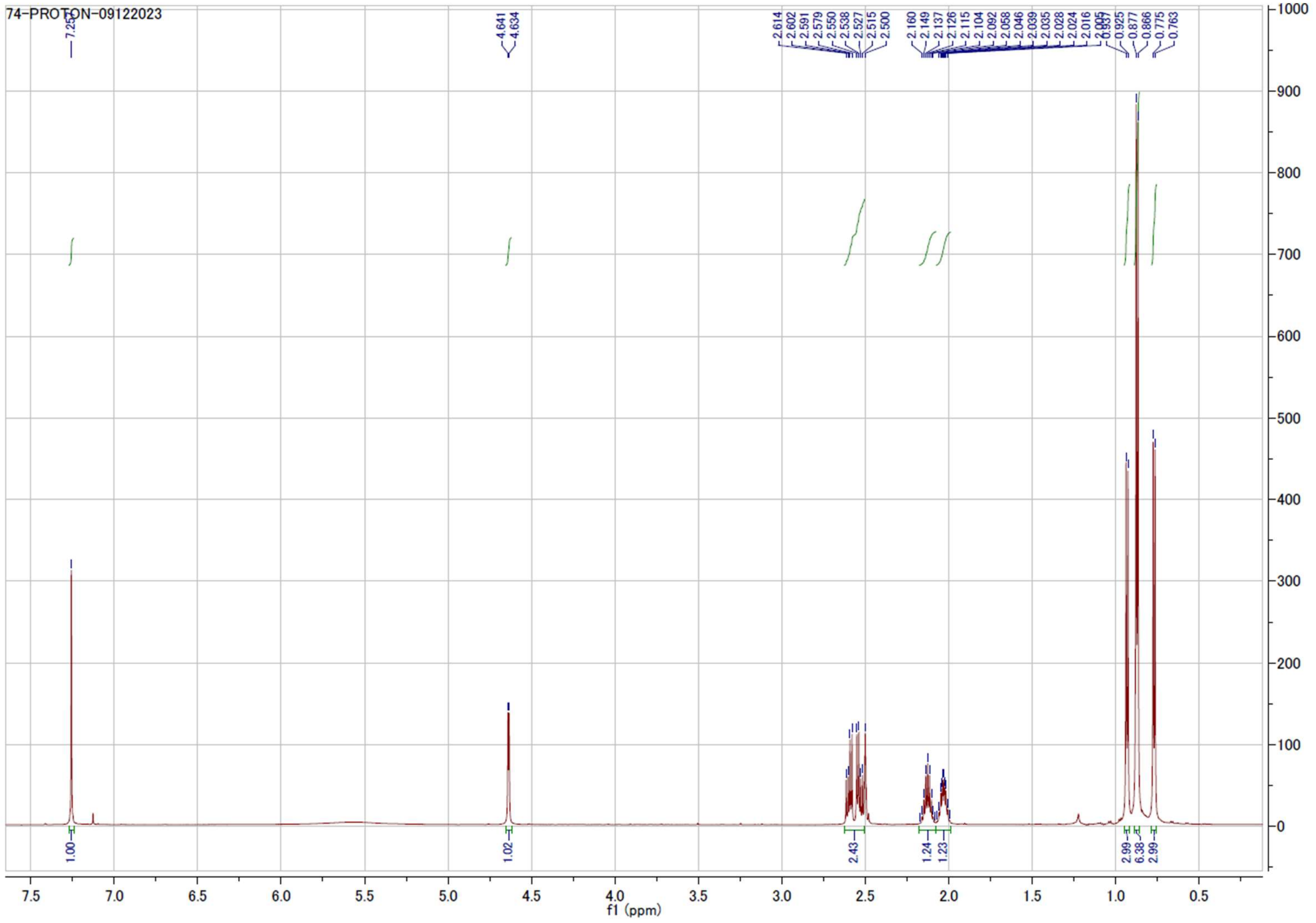

Figure S3. ^{1}H NMR spectrum of compound **1** in DMSO-*d6*

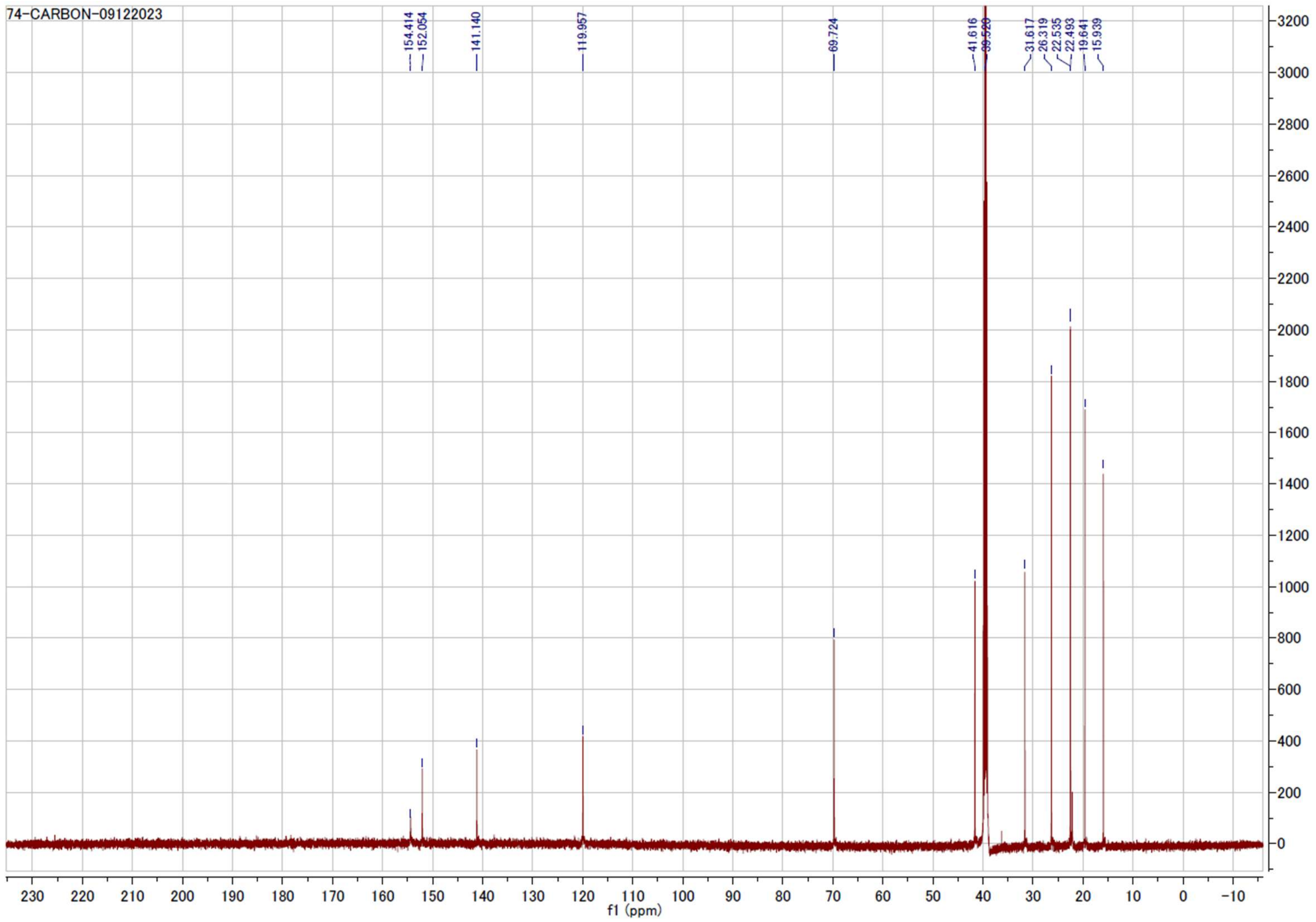

Figure S4. ^{13}C NMR spectrum of compound **1** in DMSO-*d6*

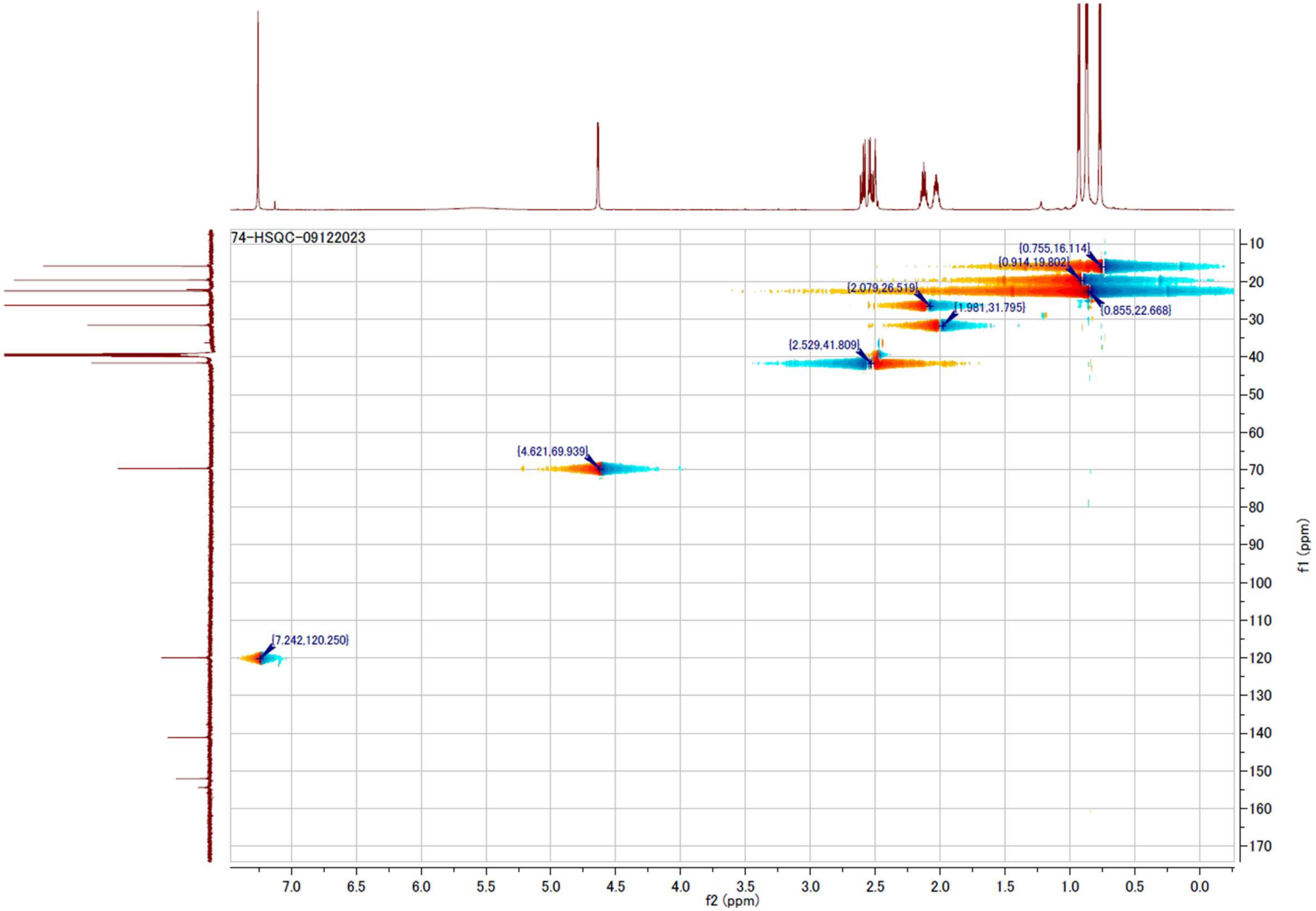

Figure S5. HSQC spectrum of compound **1** in DMSO-*d6*

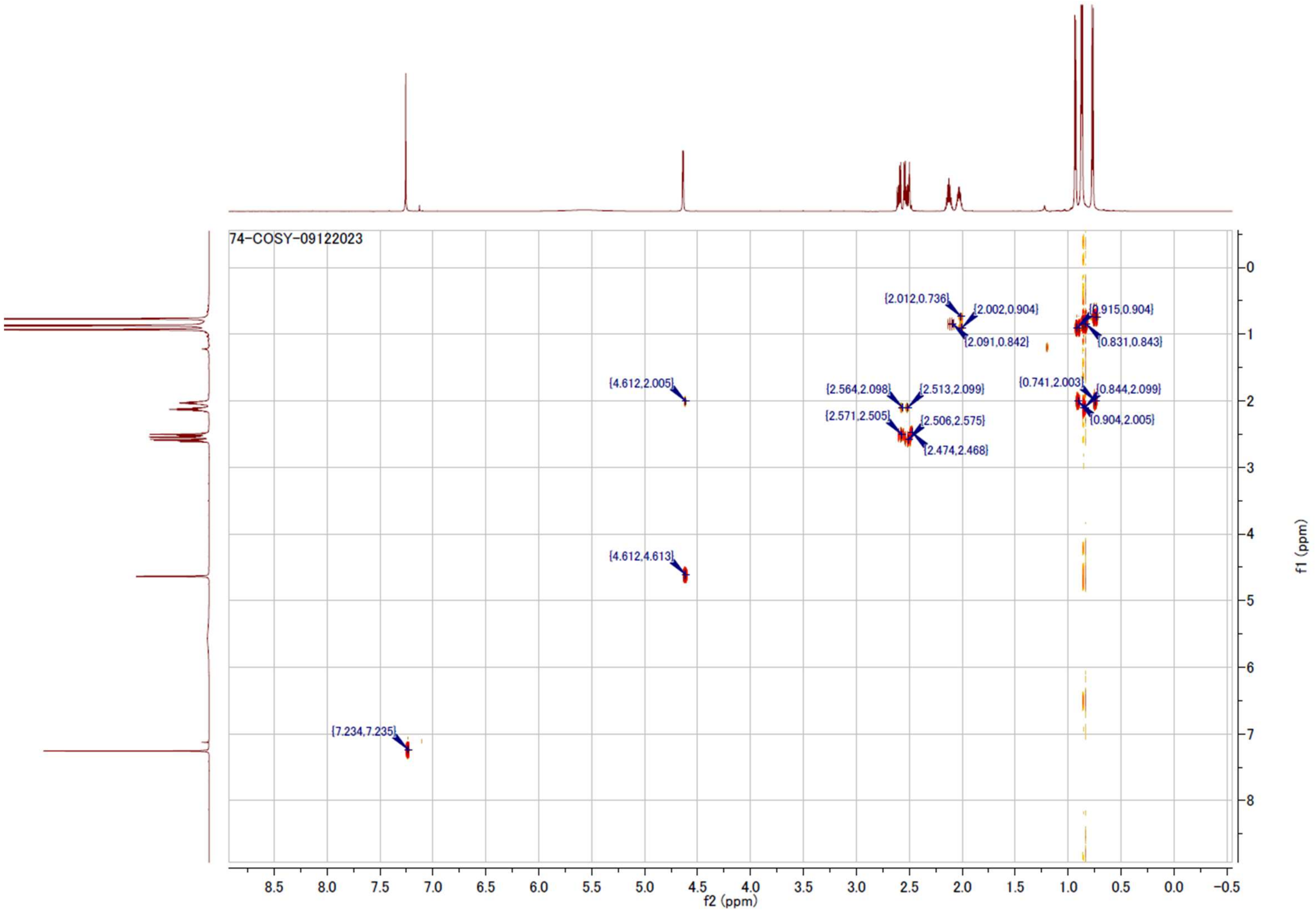

Figure S6. COSY spectrum of compound **1** in DMSO-*d6*

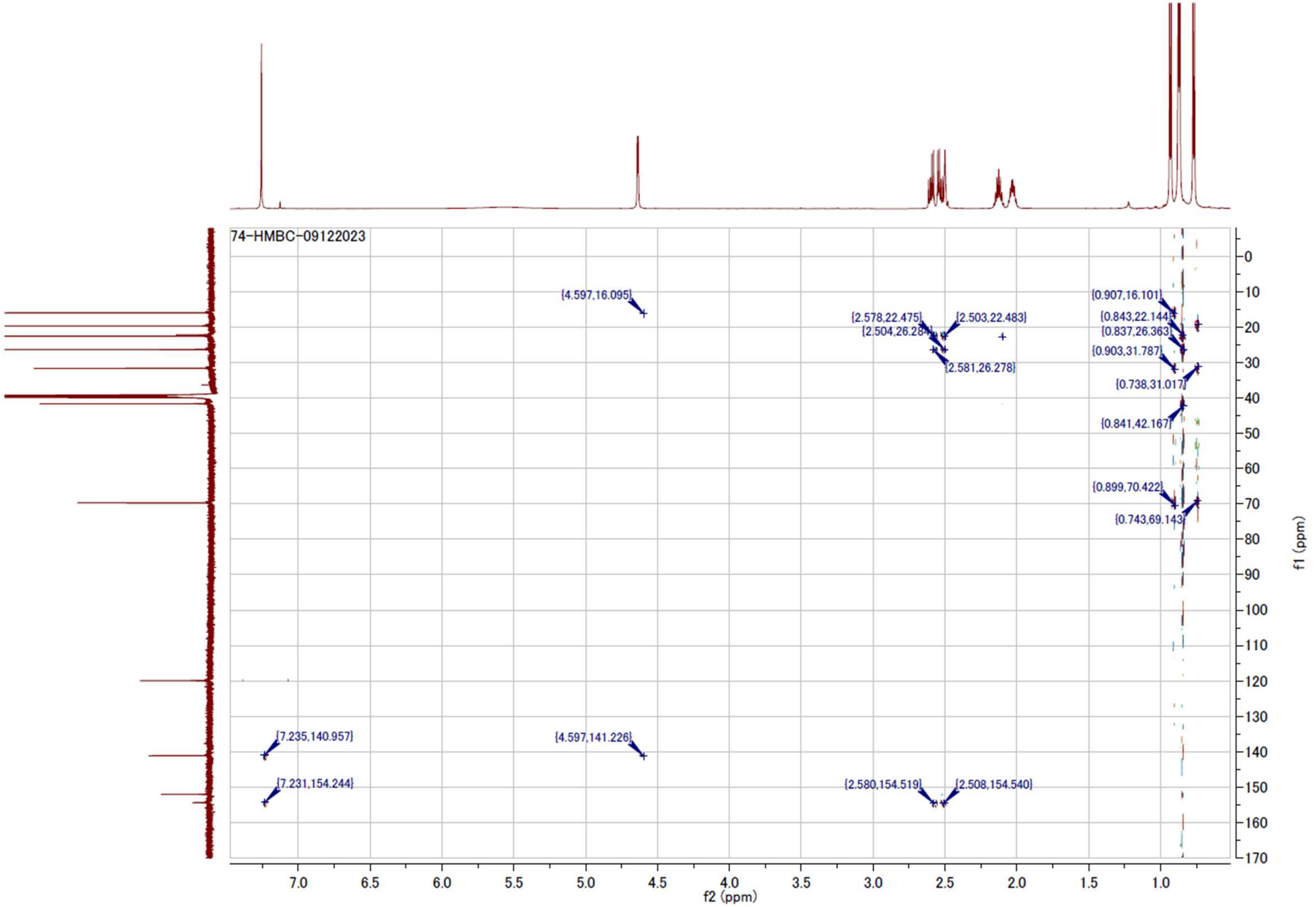

Figure S7. HMBC spectrum of compound **1** in DMSO-*d6*

Table S1. ^{1}H NMR and ^{13}C NMR comparison of compound **1** and Neohydroxyaspergillic acid (NHAA) (Zheng *et al*., 2013)

No.	Compound 1		Neohydroxyaspergillic acid (NHAA) (Zheng *et al*., 2013)	
	**δC (ppm)**	**δH (∑H, mult. *J* in Hz)**	**δC (ppm)**	**δH (∑H, mult. *J* in Hz)**
1				
2	152.0, qC		152.3, qC	
3	154.4, qC		154.5, qC	
4				
5	119.9, CH	7.26 (1H, s)	119.9, CH	7.29 (1H, s)
6	141.1, qC		141.2, qC	
1`	69.72, CH	4.64 (1H, d, *J* = 4.2)	69.8, CH	4.67 (1H, d, J = 4.2)
2`	31.6, CH	2.03 (1H, m)	31.6, CH	2.05 (1H, m)
3`	19.6, CH3	0.94 (3H, d, *J* = 7.2)	19.4, CH3	0.95 (3H, d, J = 6.8)
4`	15.9, CH3	0.77 (3H, d, *J* = 7.2)	15.9, CH3	0.79 (3H, d, J = 6.9)
1"	41.6, CH2	2.57 (2H, d, *J* = 7.0)	41.6, CH2	2.57 (2H, d, J = 7.3)
2"	26.3, CH	2.13 (1H, m)	26.3, CH	2.14 (1H, m)
3"	22.5, CH3	0.87 (3H, d, *J* = 6.6)	22.5, CH3	0.89 (3H, d, J = 6.8)
4"	22.4, CH3	0.87 (3H, d, *J* = 6.6)	22.4, CH3	0.89 (3H, d, J = 6.8)

Note :
Compound **1** : DMSO-d_6 solvent, ^{13}C NMR (150 MHz) and ^{1}H NMR (600 MHz)
Zheng *et al*., 2013 : DMSO-d_6 solvent, ^{13}C NMR (150 MHz) and ^{1}H NMR (600 MHz)

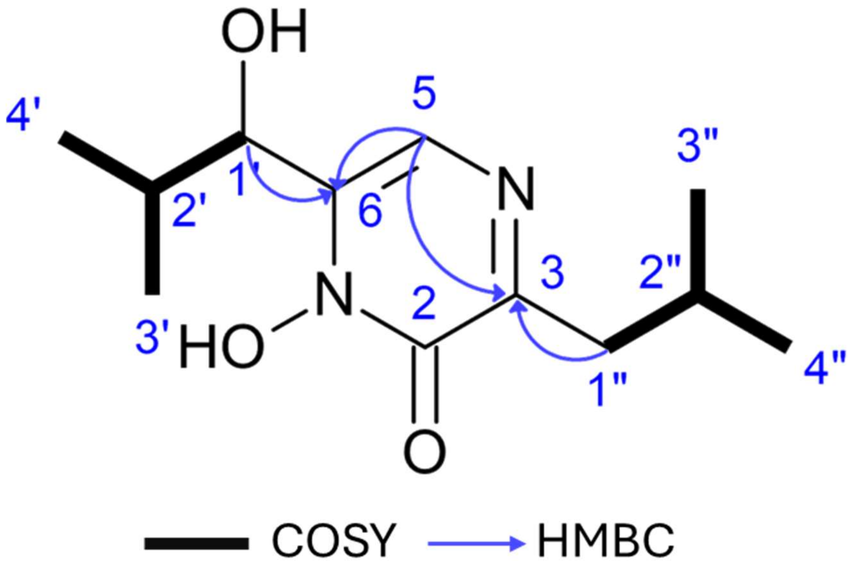

Figure S8. COSY and HMBC correlations of compound **1** (neohydroxyaspergillic acid/NHAA).

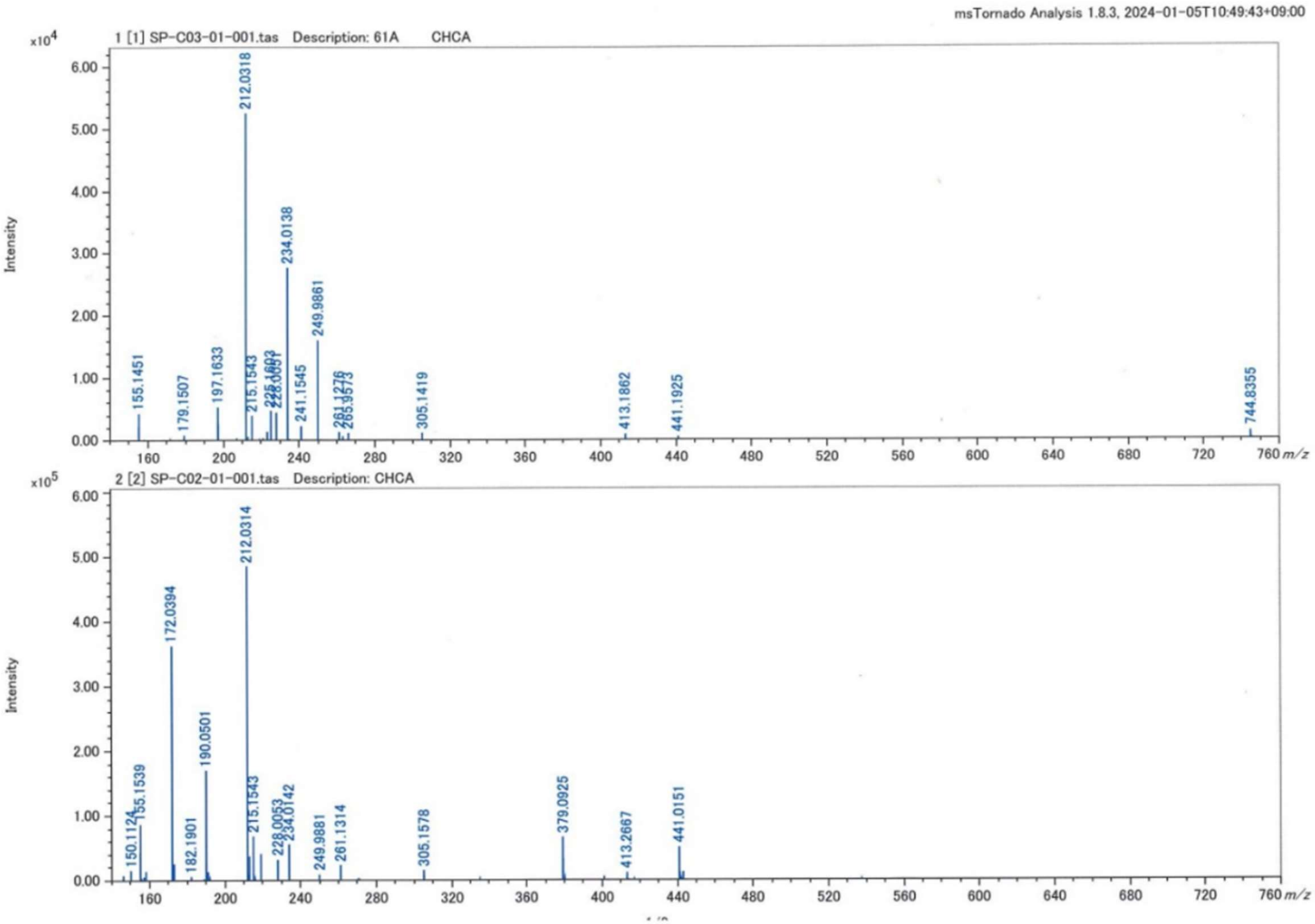

Figure S9. MALDI-TOF-MS spectrum of compound **2**

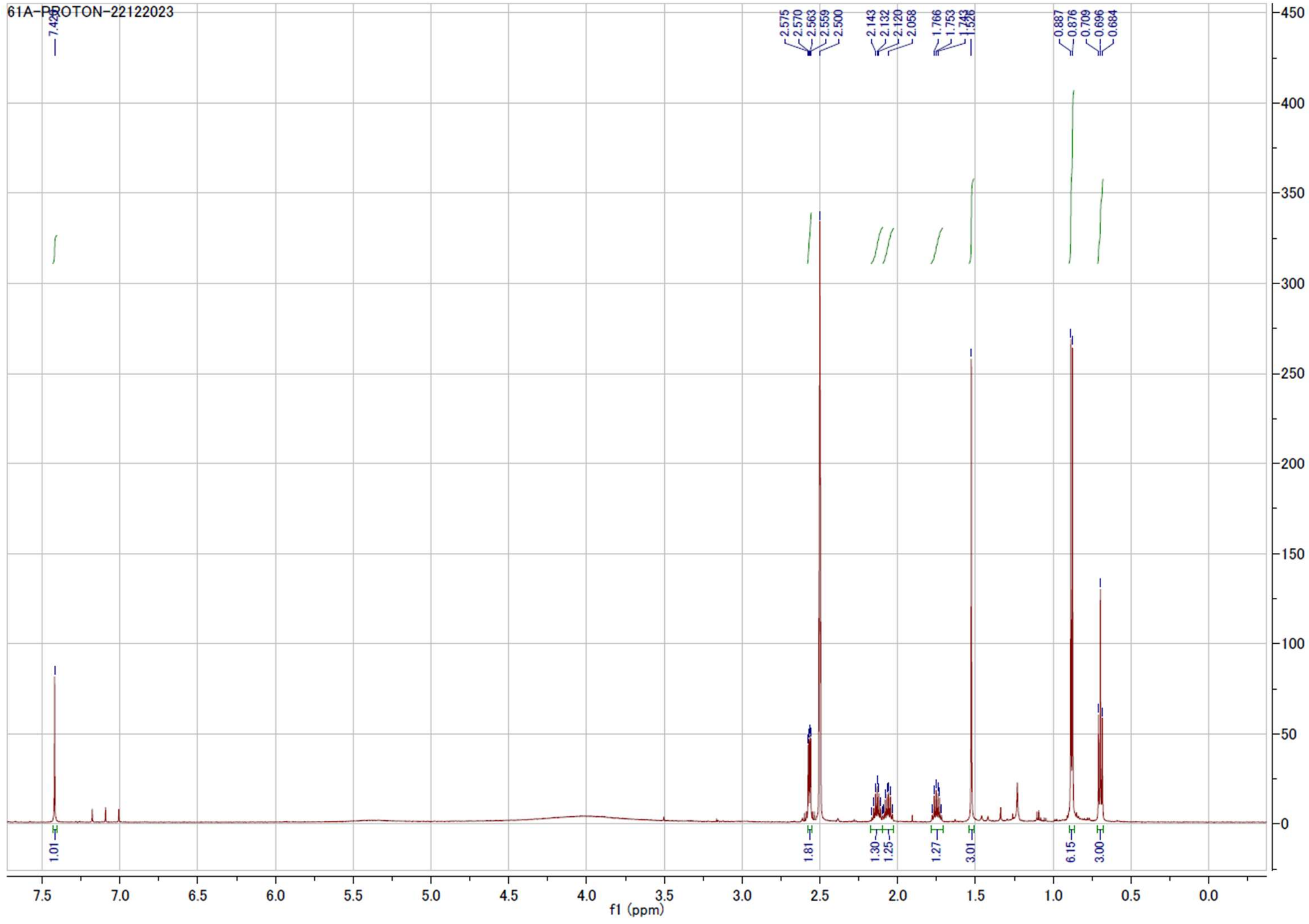

Figure S10. ^{1}H NMR spectrum of compound **2** in DMSO-*d6*

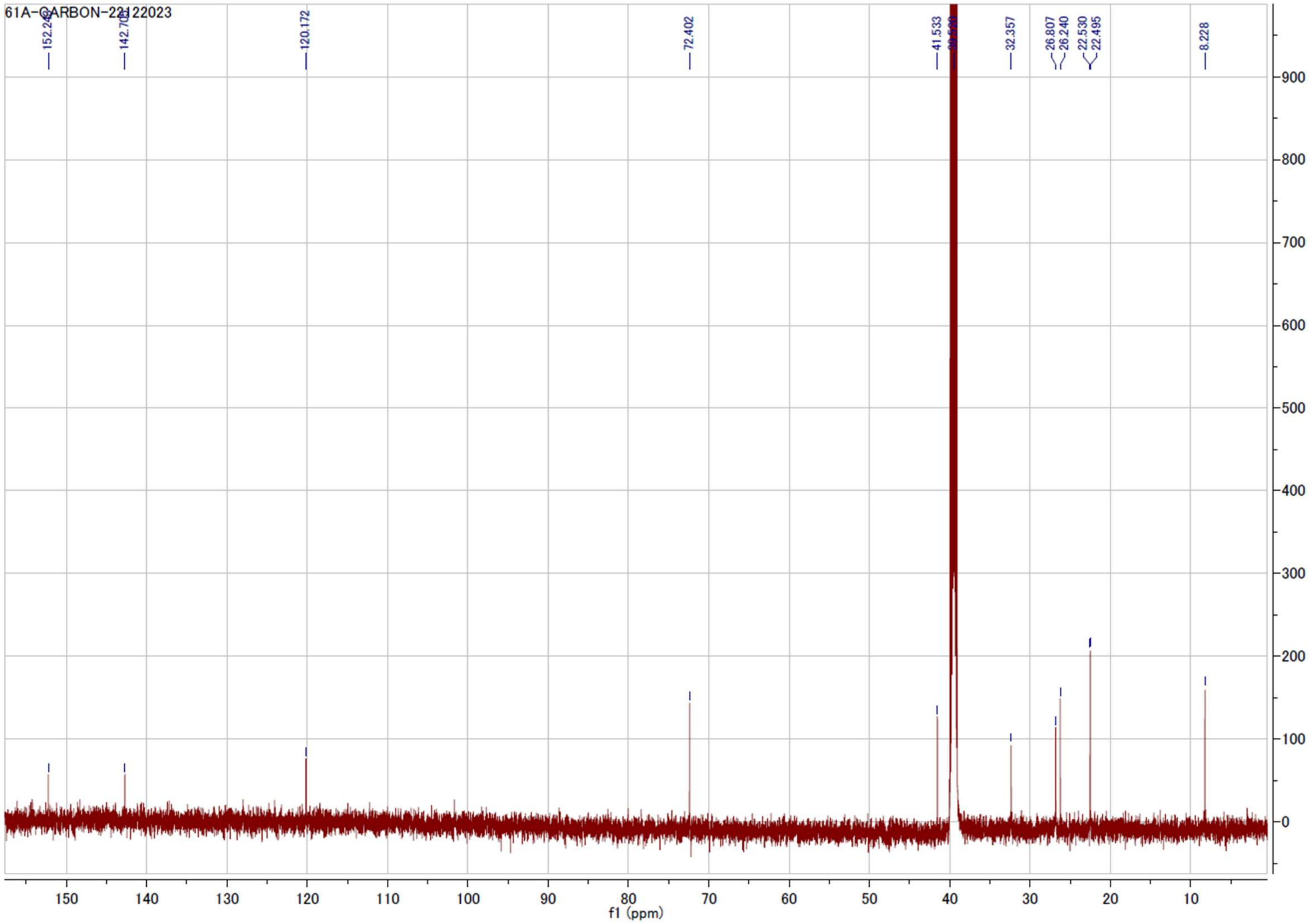

Figure S11. ^{13}C NMR spectrum of compound **2** in DMSO-*d6*

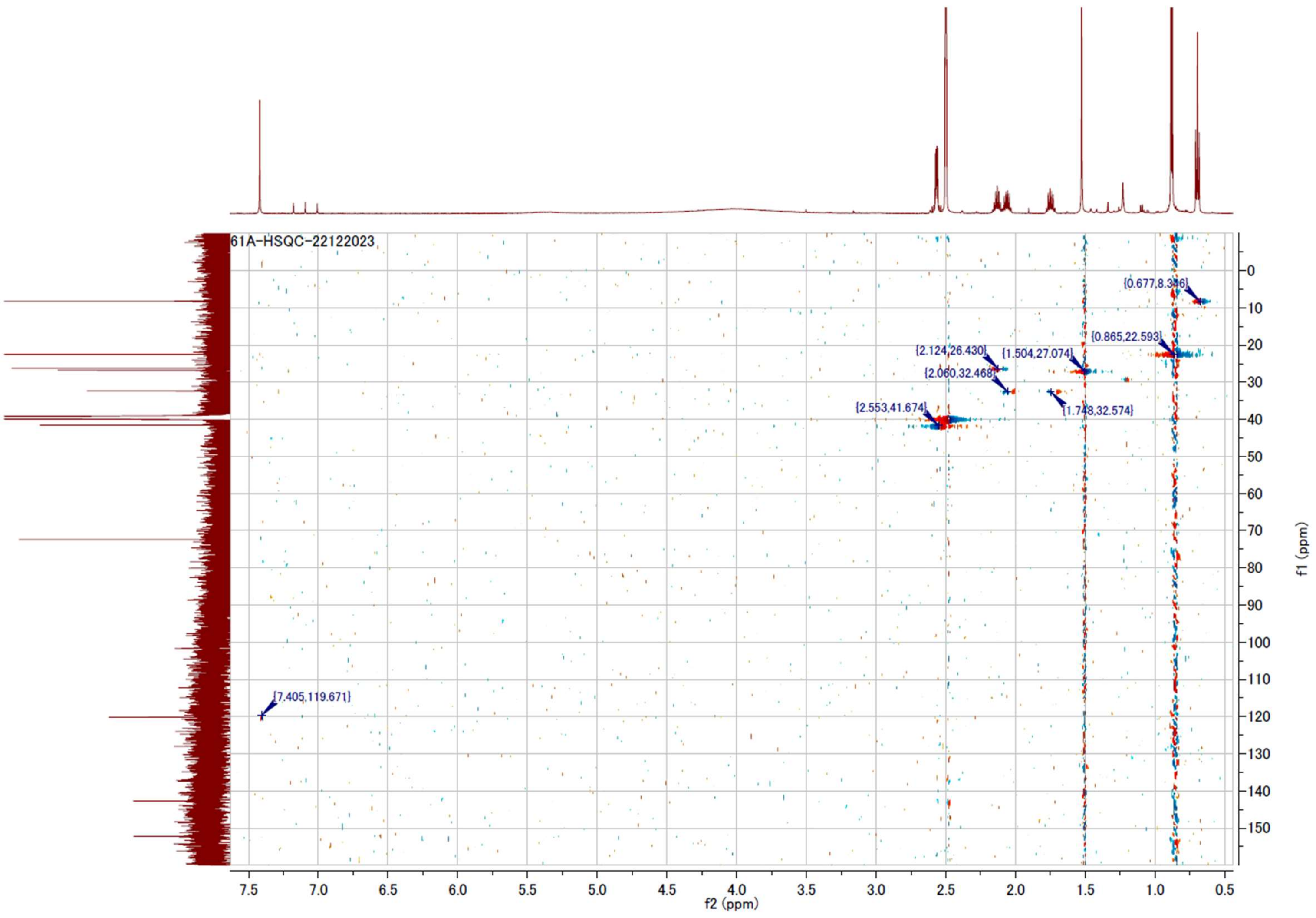

Figure S12. HSQC spectrum of compound **2** in DMSO-*d6*

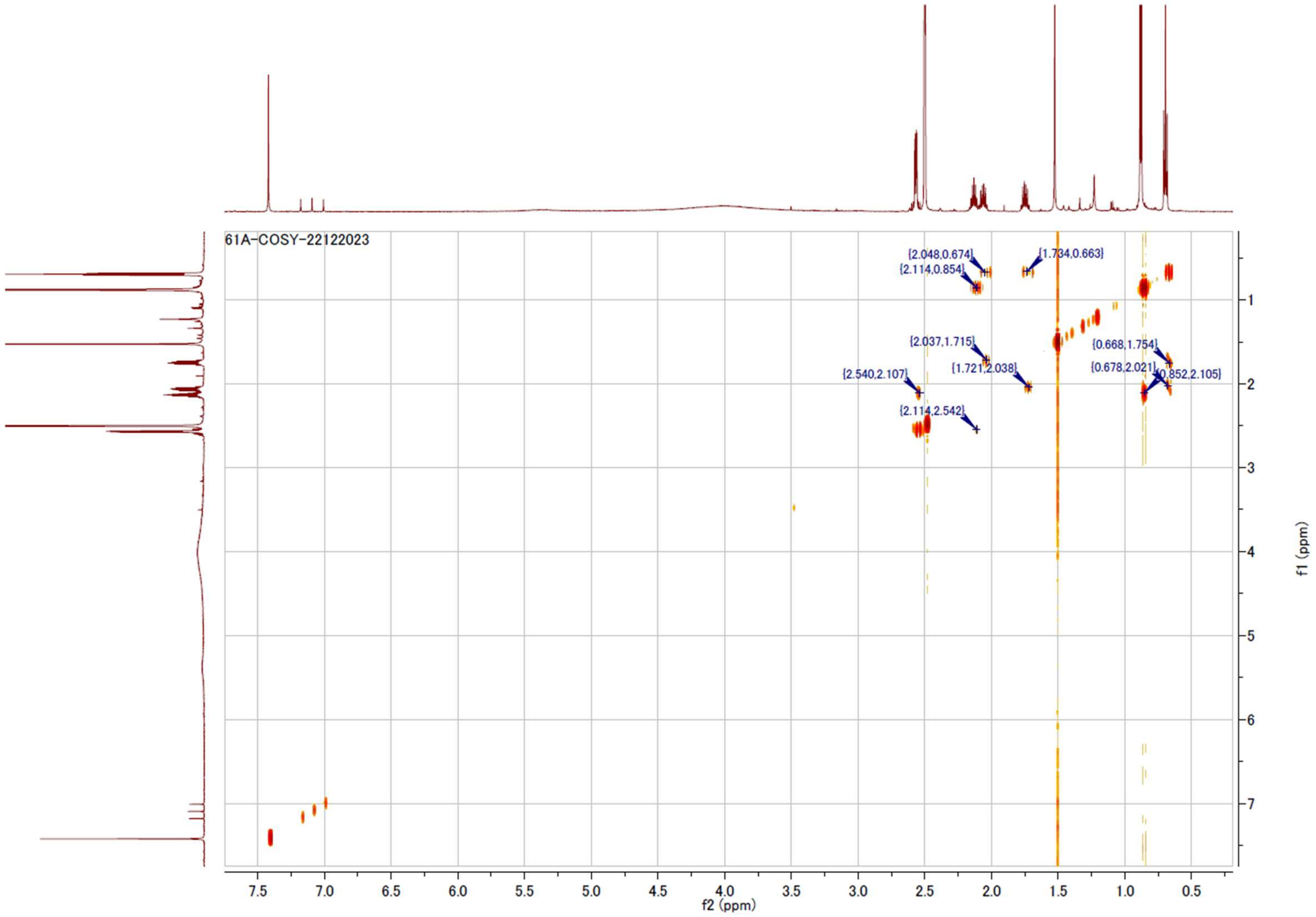

Figure S13. COSY spectrum of compound **2** in DMSO-*d6*

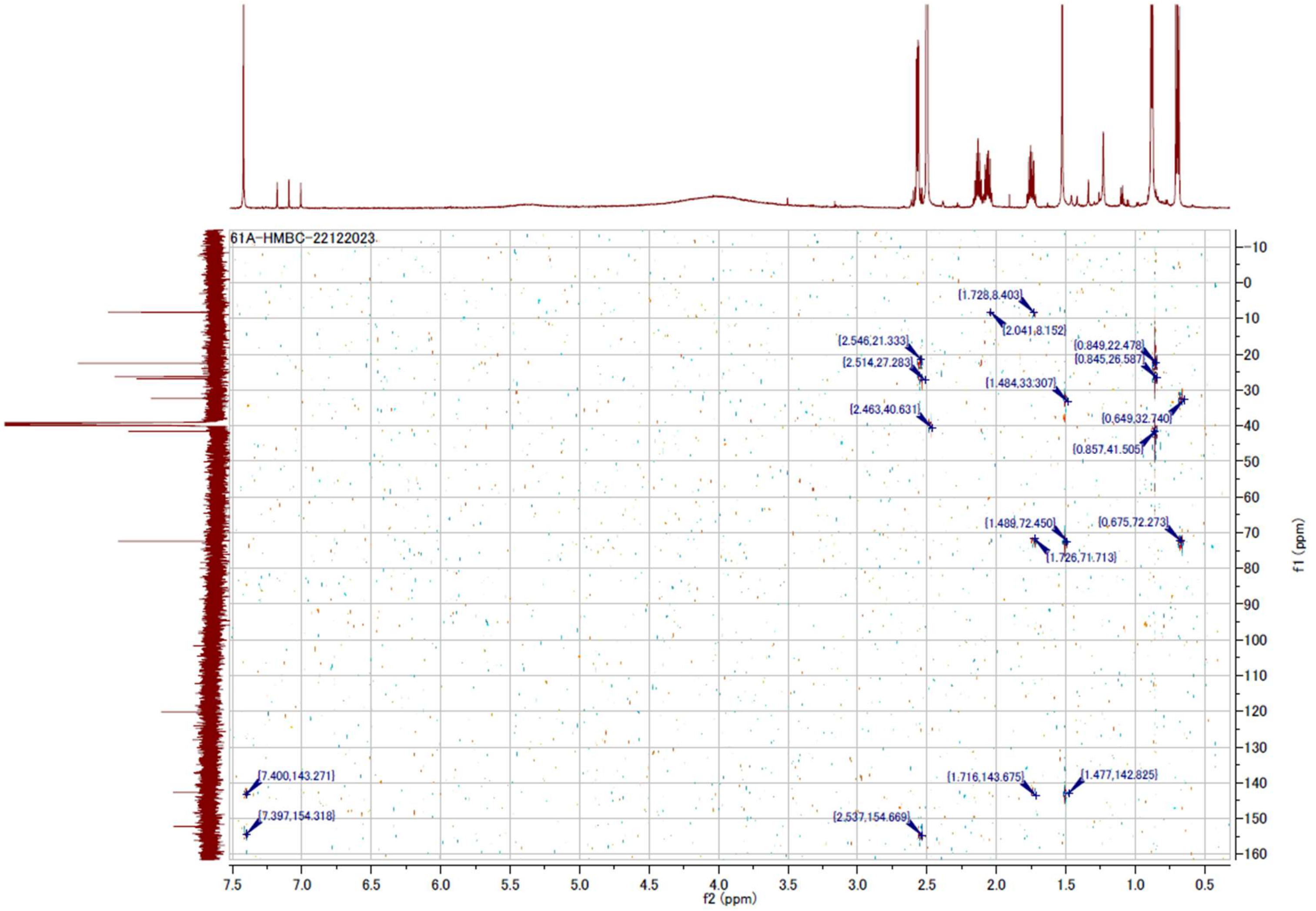

Figure S14. HMBC spectrum of compound **2** in DMSO-*d6*

Table S2. ^{1}H NMR and ^{13}C NMR comparison of compound **2** and Hydroxyaspergillic acid (HAA) (Guo *et al*., 2019).

No.	Compound 2		Hydroxyaspergillic acid (HAA) (Guo *et al*., 2019)	
	δC (ppm)	δH (∑H, mult. *J* in Hz)	δC (ppm)	δH (∑H, mult. *J* in Hz)
1				
2	152.2, qC		150.7, qC	
3	154.3, qC		155.4, qC	
4				
5	120.2, CH	7.42 (1H, s)	122.8, CH	7.73, (1H, s)
6	142.7, qC		141.7, qC	
1\`	72.4, qC		73.5, qC	
2\`	32.3, CH2	2.05 (1H, m)	32.6, CH2	1.93 (2H, m)
		1.75 (1H, m)		
3\`	26.8, CH3	1.53 (3H, s)	24.3, CH3	1.58, (3H, s)
4\`	8.2, CH3	0.69 (3H, t, *J* = 7.2)	8.6, CH3	0.80, (3H, m)
1"	41.5, CH2	2.57 (2H, m)	41.6, CH2	2.64, (2H, m)
2"	26.2, CH	2.13 (1H, m)	28.0, CH	2.01, (1H, m)
3"	22.5, CH3	0.88 (3H, d, *J* = 6.7)	22.5, CH3	0.83, (3H, m)
4"	22.4, CH3	0.88 (3H, d, *J* = 6.7)	22.5, CH3	0.83, (3H, m)

Note :
Compound 2 was recorded in DMSO-d_6 at 600 MHz for ^{1}H NMR and 150 MHz for ^{13}C NMR, whereas the comparison data from Guo et al. were recorded in $CDCl_3$ at 400 MHz for ^{1}H NMR and 100 MHz for ^{13}C NMR. Therefore, solvent- and condition-dependent chemical-shift deviations are expected. The planar structure was assigned from the present HSQC, COSY, and HMBC correlations.

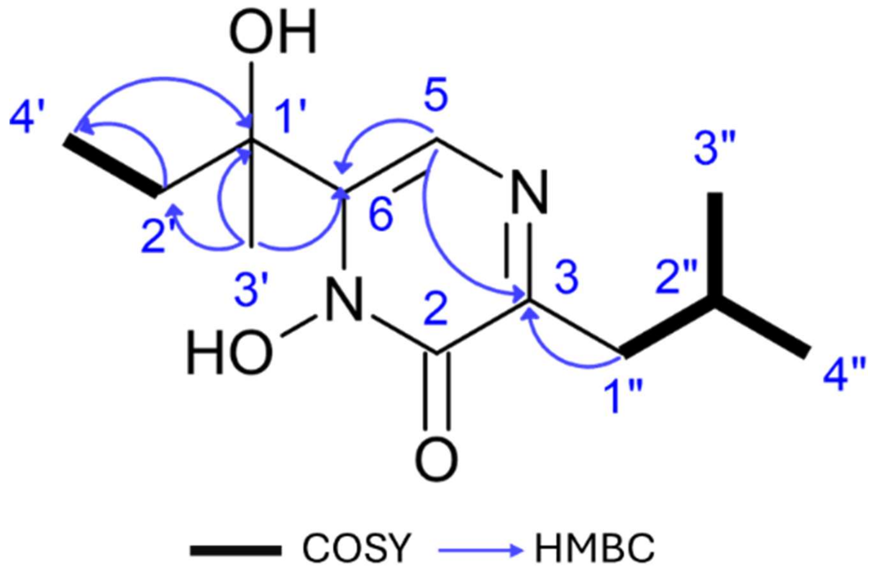

Figure S15. COSY and HMBC correlations of compound **2** (hydroxyaspergillic acid/HAA).

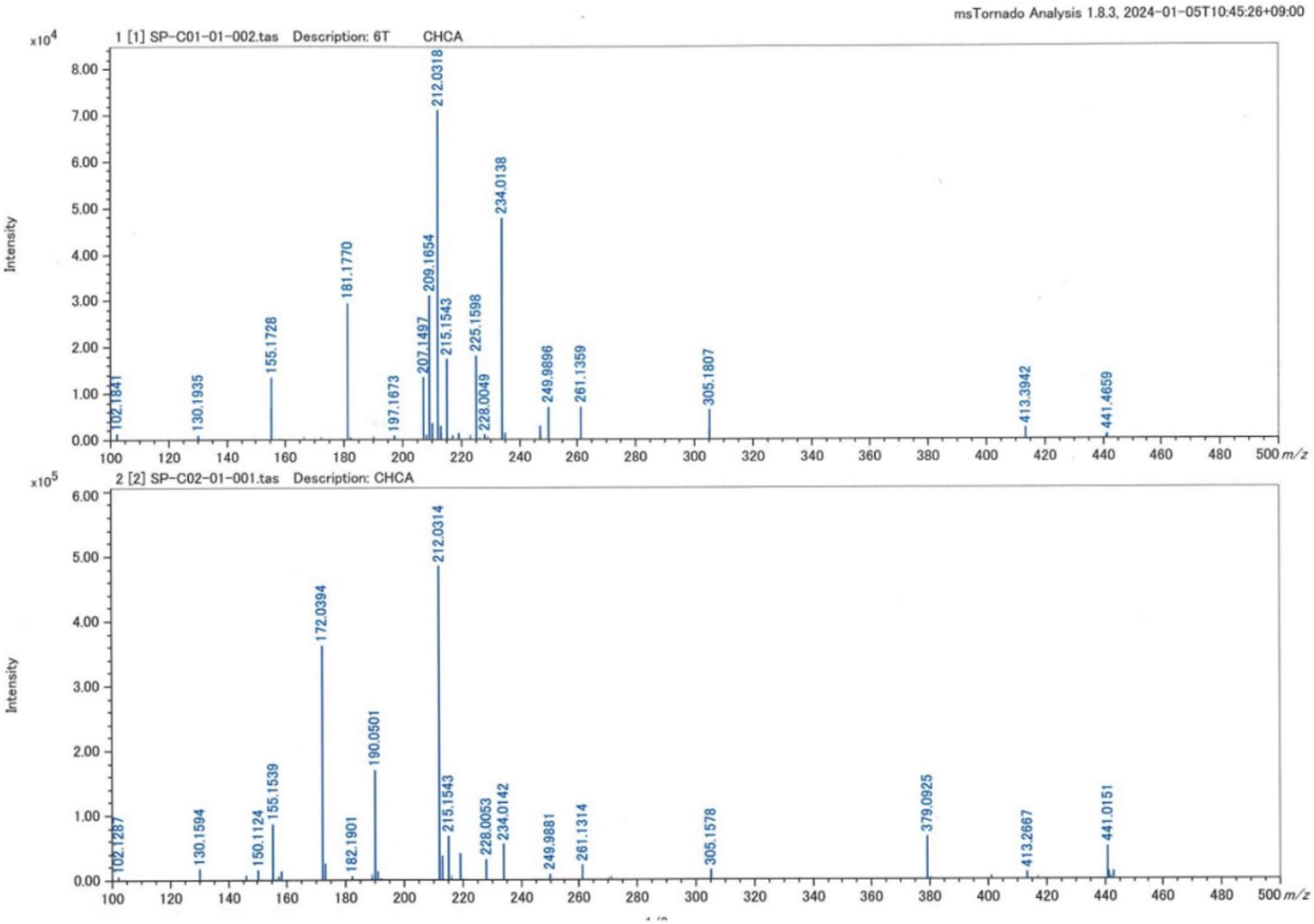

Figure S16. MALDI-TOF-MS spectrum of compound **3**

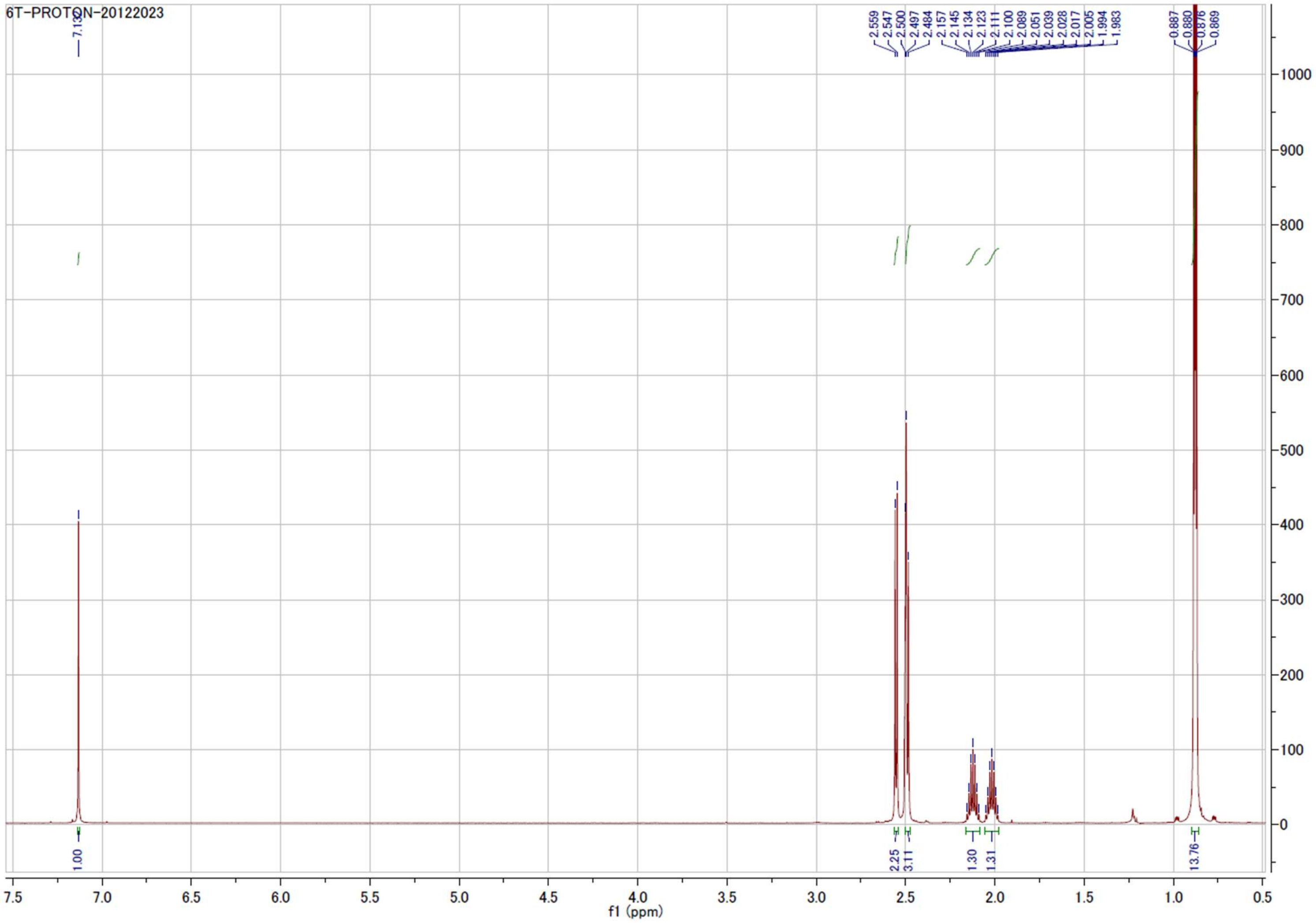

Figure S17. ^{1}H NMR spectrum of compound **3** in DMSO-*d6*

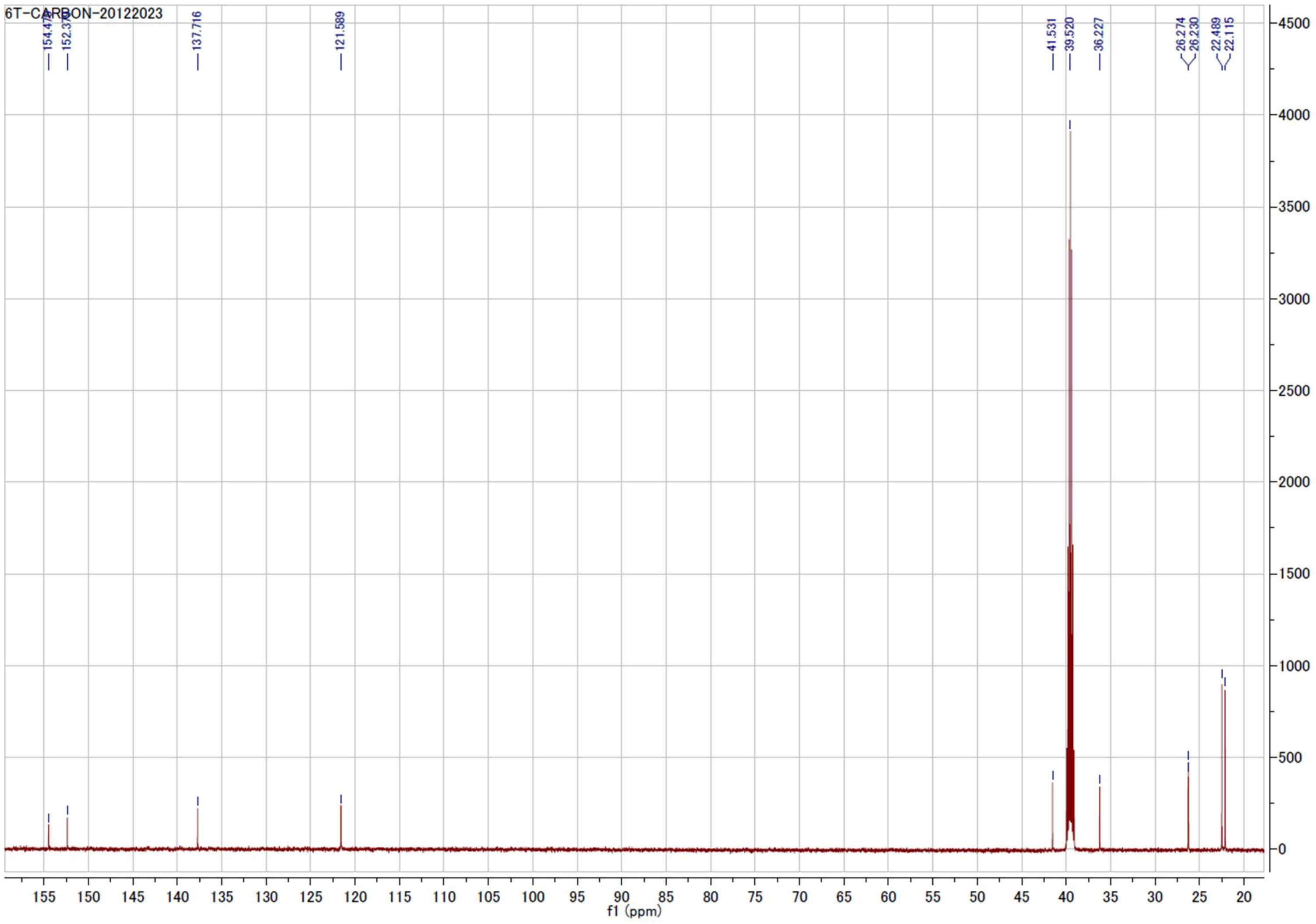

Figure S18. ^{13}C NMR spectrum of compound **3** in DMSO-*d6*

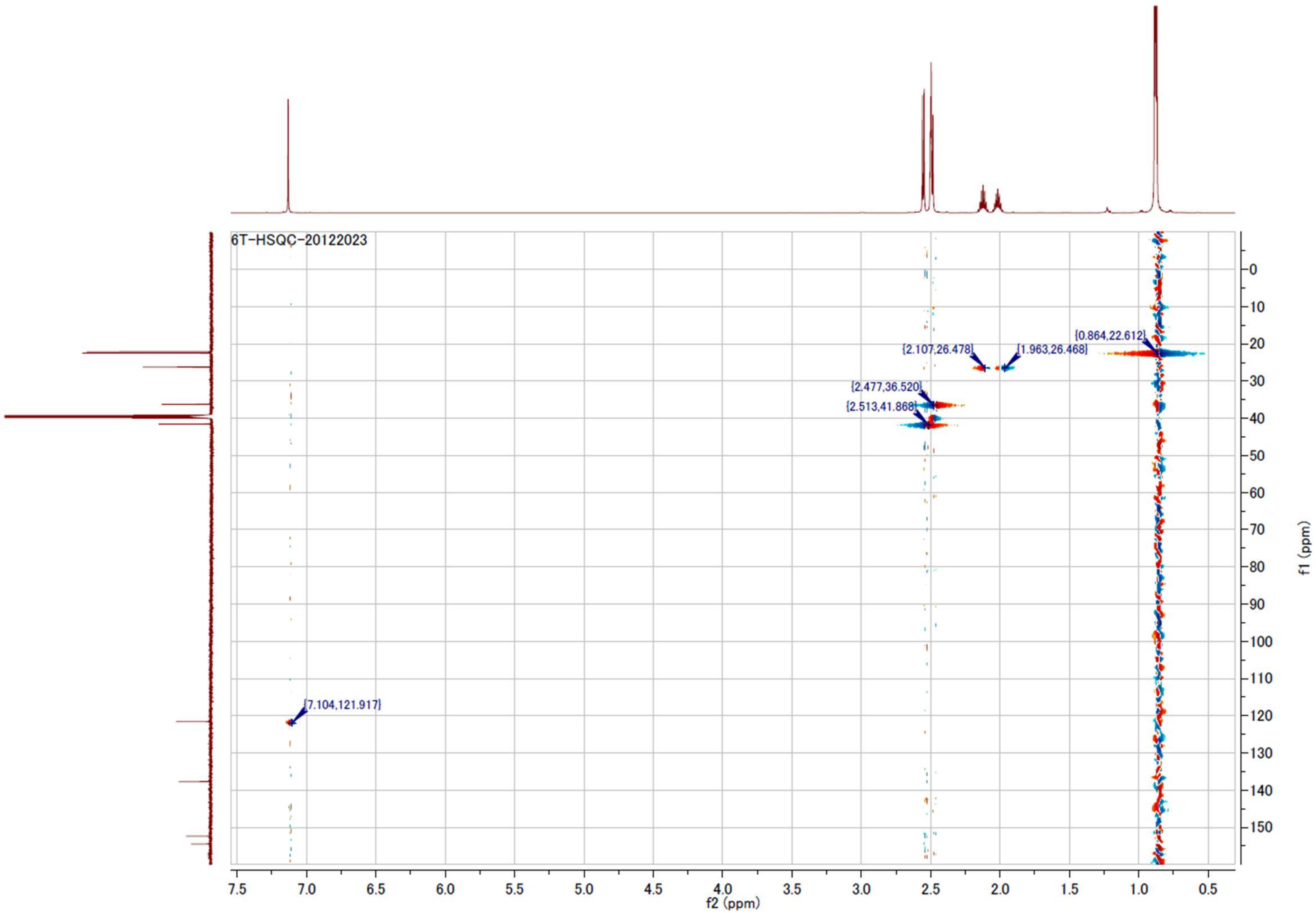

Figure S19. HSQC spectrum of compound **3** in DMSO-*d6*

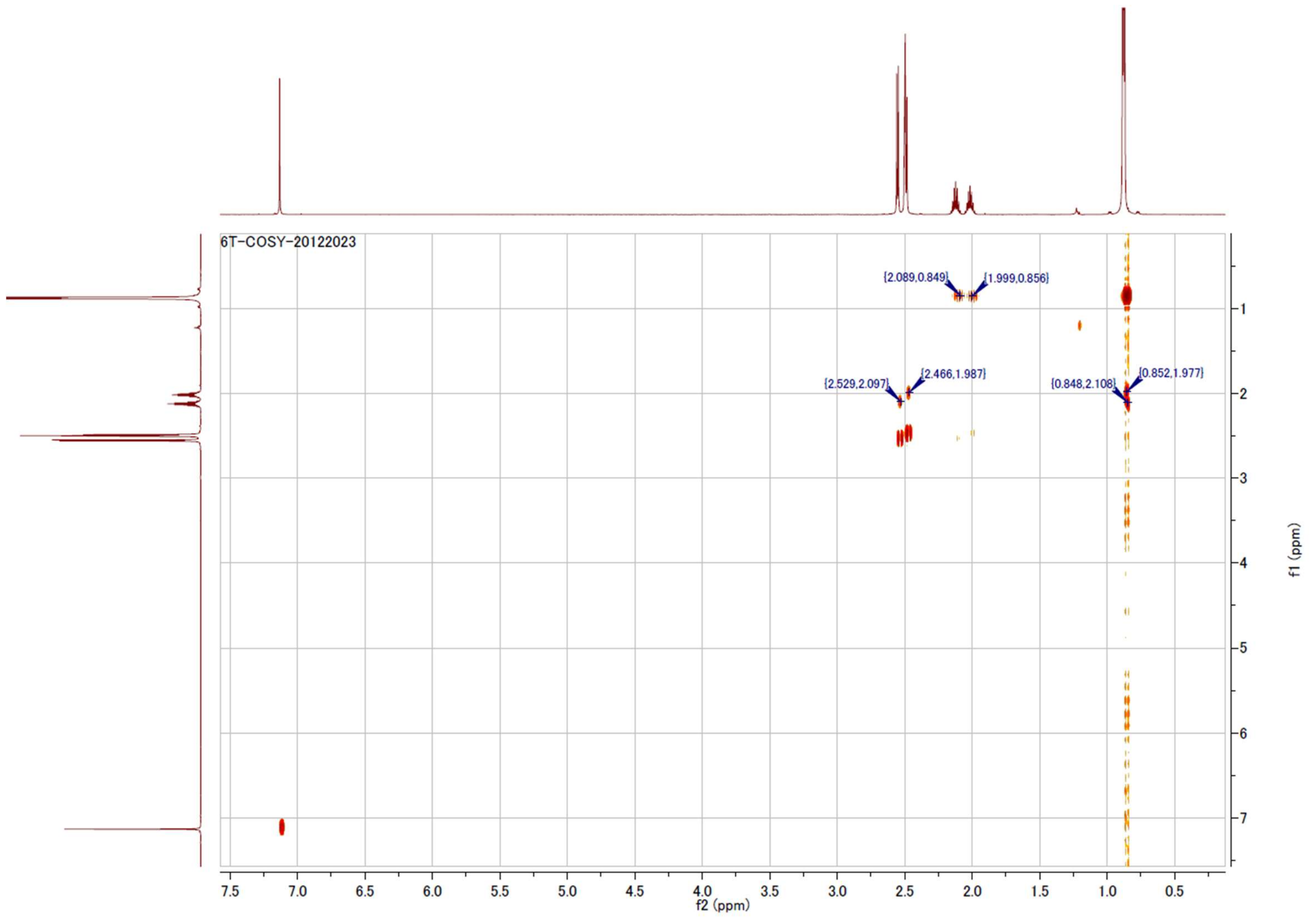

Figure S20. COSY spectrum of compound **3** in DMSO-*d6*

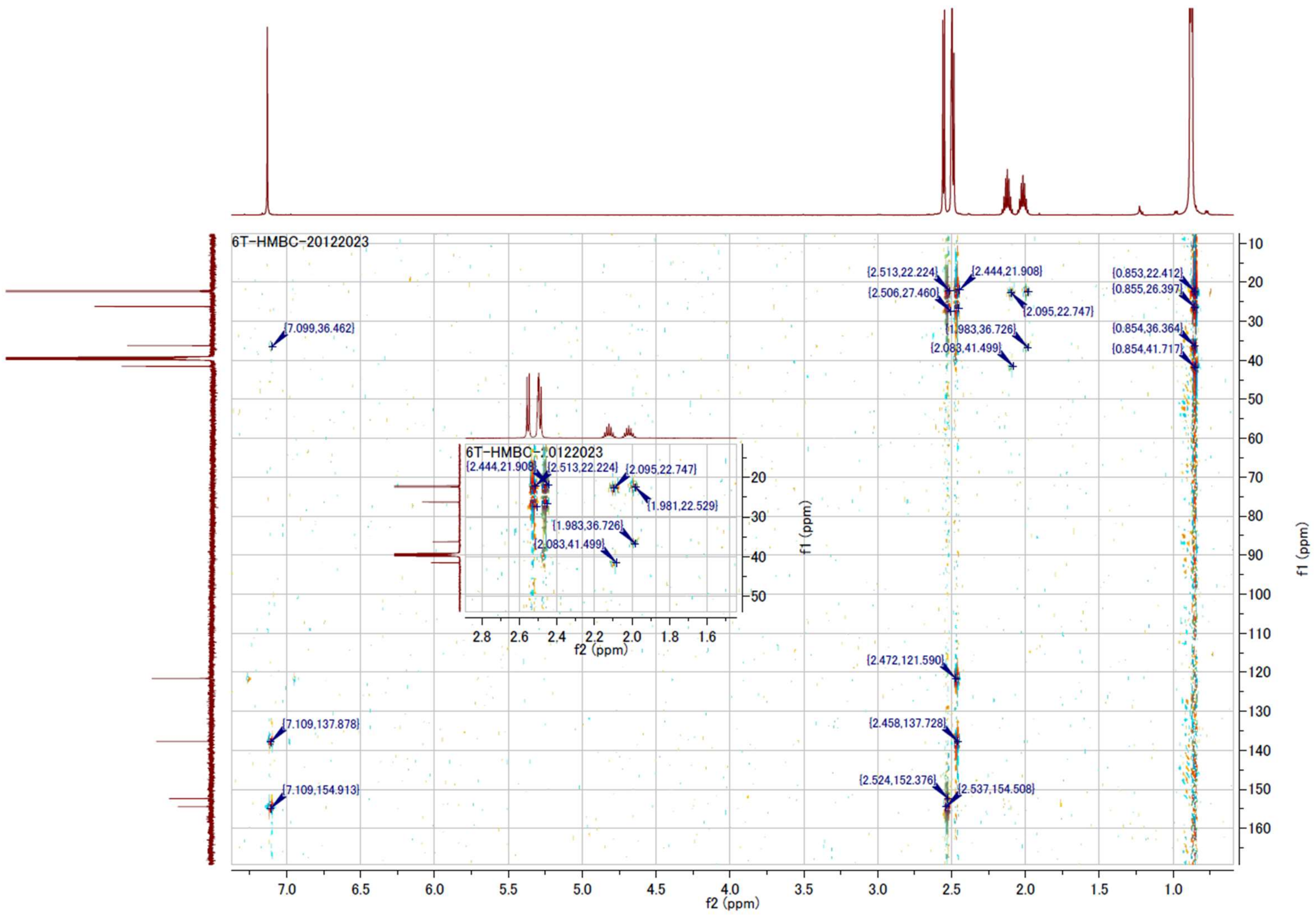

Figure S21. HMBC spectrum of compound **3** in DMSO-*d6*

Table S3. ^{1}H NMR and ^{13}C NMR comparison of compound **3** and Neoaspergillic acid (NAA) (Zheng *et al*., 2013)

No.	Compound 3		Neoaspergillic acid (Zheng *et al.*, 2013)	
	δC (ppm)	δH (∑H, mult. *J* in Hz)	δC (ppm)	δH (∑H, mult. *J in* Hz)
1				
2	152.4, qC		152.4, qC	
3	154.5, qC		154.4, qC	
4				
5	121.6, CH	7.13 (1H, s)	121.6, CH	7.15 (1H, s)
6	137.7, qC		137.7, qC	
1`	36.2, CH2	2.51 (2H, d, *J* = 7.2)	36.0, CH2	2.51 (2H, d, *J* = 7.3)
2`	26.3, CH	2.02 (1H, m)	26.1, CH	2.04 (1H, m)
3`	22.1, CH3	0.87 (3H, d, *J* = 4.2)	19.4, CH3	0.89 (3H, d, *J* = 4.1)
4`	22.1, CH3	0.87 (3H, d, *J* = 4.2)	19.4, CH3	0.89 (3H, d, *J* = 4.1)
1"	41.5, CH2	2.56 (2H, d, *J* = 7.2)	41.7, CH2	2.57 (2H, d, *J* = 7.3)
2"	26.2, CH	2.12 (1H, m)	26.2, CH	2.14 (1H, m)
3"	22.5, CH3	0.88 (3H, d, *J* = 4.2)	19.5, CH3	0.91 (3H, d, *J* = 4.1)
4"	22.5, CH3	0.88 (3H, d, *J* = 4.2)	19.5, CH3	0.91 (3H, d, *J* = 4.1)

Note :
Compound **3** : DMSO-d_6 solvent, ^{13}C NMR (150 MHz) and ^{1}H NMR (600 MHz)
Zheng *et al*., 2013 : DMSO-d_6 solvent, ^{13}C NMR (150 MHz) and ^{1}H NMR (600 MHz)
Compound 3 was assigned as neoaspergillic acid based on MS, 1D/2D NMR correlations, and comparison with reported data. The discrepancy relative to Zheng et al. is confined to the aliphatic methyl carbon region. The pyrazinone core, methylene, and methine signals agree closely with the reported data. The methyl proton signals at δH 0.87–0.88 directly correlated with methyl carbons at δC 22.1 and 22.5 in the present HSQC spectrum; therefore, the methyl carbon assignments were retained according to the present HSQC/HMBC data.

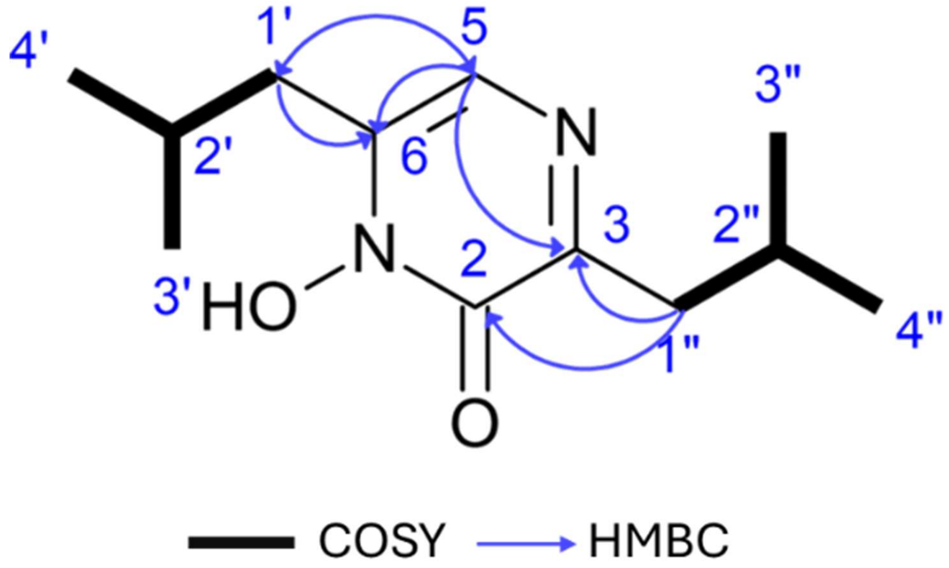

Figure S22. COSY and HMBC correlations of compound **3** (Neoaspergillic acid/NAA).
